# TLR7-mediated autoimmunity targets Robo3 to drive astrogliosis in frontotemporal dementia

**DOI:** 10.21203/rs.3.rs-11236688/v1

**Published:** 2026-10-07

**Authors:** Rufei Lu, Jennifer Ja-Yoon Choi, Naznin Jahan, Isabella Imhof, Samuel Collo, Sofia Bustamante Eguiguren, Jiasheng Zhang, Gavin M. Sowa, Colin R. Zamecnik, Juliet K. Chen, Jeffrey W. Hofmann, Mary Nakamura, Michael R. Wilson, Raymund Bueno, Yang Sun, Chun Jimmie Ye, Christine L. Hsieh, Eric J. Huang

**Affiliations:** 1 Department of Pathology, University of California San Francisco, San Francisco, CA 94143, U.S.A.; 2 Weill Institute for Neurosciences, University of California San Francisco, San Francisco, CA 94158, U.S.A.; 3 Arthritis and Clinical Immunology Research Program, Oklahoma Medical Research Foundation, Oklahoma City, OK 73104, U.S.A.; 4 BMS Graduate Program, University of California San Francisco, San Francisco, CA 94143, U.S.A.; 5 Division of Rheumatology, Department of Medicine, University of California San Francisco, San Francisco, CA 94143, U.S.A.; 6 Veterans Administrations Health Care System, San Francisco, CA 94121; 7 Department of Neurology, University of California San Francisco, San Francisco, CA 94143, U.S.A.; 8 Department of Pathology and Immunology, Washington University School of Medicine, St. Louis, MO 63110, U.S.A.; 9 These authors contributed equally

**Keywords:** Progranulin, C9orf72, Frontotemporal dementia, Autoimmunity, Age-associated B cells, Clonal expansion, Robo3, Astrocytes, Neuroinflammation, Neurodegeneration

## Abstract

Mutations in *GRN* and *C9orf72* are common causes of frontotemporal dementia (FTD). While it is well-recognized that *GRN* or *C9orf72* mutations also increase the risk of autoimmune diseases, the intersection of innate and adaptive immunity, neuroinflammation, and neurodegeneration in FTD remains unclear. Here we show that concurrent loss of *Grn* and *C9orf72* breaks immune tolerance by activating the pattern recognition receptor, TLR7, leading to clonal expansion of age-associated B cells and activated B cells. *Grn*^*−/−*^*;C9orf72*^*−/−*^ B cells exhibited heightened responses upon TLR7 activation and produced autoantibodies that target the axon guidance receptor Robo3 to promote astroglial pathology. Interestingly, immunization with Robo3 extracellular domain in young *Grn*^*−/−*^*;C9orf72*^*−/−*^ mice accelerated astrogliosis. Conversely, TLR7 antagonist M5049 treatment rescued B cell defects, reduced anti-Robo3 autoantibodies, and mitigated astrogliosis and synaptic loss in *Grn*^*−/−*^*;C9orf72*^*−/−*^ mice. Together, these results offer critical insights into how TLR7-mediated autoimmune dysfunction targets Robo3 to drive neuroinflammation and neurodegeneration in FTD.

Mutations in *Progranulin* (*GRN*) and *C9orf72* are common causes of frontotemporal dementia (FTD)^1–4^, which is a major cause of dementia in patients under 65 years old. The clinical manifestations in FTD patients include progressive behavioral changes, deterioration in language skills, and movement disorders^5^. In case-control studies, *GRN* or *C9orf72* mutation carriers show a higher propensity to develop autoimmune diseases, whereas *GRN* and *C9orf72* dual mutation carriers manifest early disease-onset and severe autoimmune dysfunction, including inflammatory arthritides, cutaneous disorders, and celiac disease^6–8^. Furthermore, co-occurrence of *C9orf72* and *GRN* mutations in the same families increases the risk to develop severe autoimmune conditions and early-onset neurodegeneration^8,9^. Finally, several known autoimmune diseases have abnormalities in *GRN* and/or *C9orf72*. For instance, polymorphisms in the 3’ untranslated region (UTR) of *C9orf72* influence the response of patients with rheumatoid arthritis (RA) to anti-TNF treatment^10^. Patients with systemic lupus erythematosus (SLE) and rheumatoid arthritis (RA) have a high propensity to carry intermediate GGGGCC expansion repeats (9–30) in *C9orf72*^11^.

Mechanistically, Progranulin and its cleavage products granulins have been implicated in regulating endolysosomal trafficking in microglia^12^. As such, loss-of-function mutations in Progranulin lead to age-dependent microgliosis with prominent accumulation of lysosomes in Progranulin-deficient reactive microglia^13,14^. In parallel, C9orf72 has been implicated in the initiation of autophagy^15,16^, recycling of endosomes, and lysosomal biogenesis in motor neurons^17^. In addition, C9orf72-deficient myeloid cells and dendritic cells are hypersensitive to activators of the stimulator of interferon genes (STING) signaling pathway^18^. Despite the robust evidence supporting C9orf72 and Progranulin in innate immune responses, it remains unclear whether and how autoimmune dysfunction caused by haploinsufficiency in both *GRN* and *C9orf72* might promote neuroinflammation and neurodegeneration.

Here, we show that deficiency in both Progranulin and C9orf72 promotes autoimmunity via TLR7-mediated clonal expansion of B cells, which produce autoantibodies that target the axon guidance protein Robo3 in astrocytes to promote neuroinflammation and neurodegeneration. These results provide new insights into mechanisms of autoimmunity in FTD and offer critical implications for potential therapies.

## RESULTS

### Aberrant B cell expansion in *Grn*^*−/−*^*;C9orf72*^*−/−*^ mice

To investigate how loss-of-function in *GRN* and *C9orf72* could synergistically promote autoimmunity and neuroinflammation, we first generated an aging cohort of mice with loss of *Gm*, *C9orf72*, or both genes. Kaplan-Meier survival curves revealed that *Grn*^*−/−*^*;C9orf72*^*−/−*^ mice exhibited early lethality (median survival 442 days) compared to *Grn*^*−/−*^ (520 days) and *C9orf72*^*−/−*^ mice (645 days) and *wild type* mice (735 days) (**Extended Data Fig. 1a**). At necropsy, *Grn*^*−/−*^*;C9orf72*^*−/−*^ mice showed more severe cervical lymphadenopathy, splenomegaly, and glomerulonephritis at 7 and 12 months than *C9orf72*^*−/−*^ or *Grn*^*−/−*^ mice, with a significant effacement of the lymphoid architecture in the spleen and increased IgG deposits in the glomeruli in kidneys (**Extended Data Fig. 1b-g**). Compared to other genotypes, *Grn*^*−/−*^*;C9orf72*^*−/−*^ mice also had elevated anti-dsDNA and anti-nuclear antibodies (ANA) in sera, though no drastic increases in IgG or IgA levels (**Extended Data Fig. 1h-i**).

To characterize the peripheral immune dysfunction in *Grn*^*−/−*^*;C9orf72*^*−/−*^ mice, we used cellular indexing of transcriptomes and epitopes by sequencing (CITE-seq)^19,20^ with cell hashing to simultaneously analyze single-cell transcriptomes and 138 cell surface proteins in splenocytes from 7-month-old *wild type*, *Grn*^*−/−*^, *C9orf72*^*−/−*^, and *Grn*^*−/−*^*;C9orf72*^*−/−*^ mice (**Extended Data Fig. 2a-e**, **Supplementary Table 1**). Visualization of immune cell subsets by uniform manifold approximation and projection (UMAP) indicated a significant expansion of age-associated B cells (ABCs) (c19), IgM^−^ IgD^−^ (double negative or DN) B cells (c20), and activated B cells (c21) in *Grn*^*−/−*^*;C9orf72*^*−/−*^ mice compared to other genotypes (Fig. 1a, **arrowheads**). Analyses of the B cell populations further showed that a large proportion of activated B cells (CD80/CD86^hi^ and MHCII^hi^) expressed *Tbx21*, *Itgam*, and *CD80*, which in pseudotime analysis were downstream of the differentiation trajectories of ABCs^21,22^ (Fig. 1b–c, **Extended Data Fig. 2f**). Compared to *wild type*, *C9orf72*^*−/−*^, and *Grn*^*−/−*^ mice, splenocytes in *Grn*^*−/−*^*;C9orf72*^*−/−*^ mice showed not only a significant expansion of ABCs, IgM^−^ IgD^−^ B cells, and activated B cells, but also a significant reduction of marginal zone (MZ) B cells. Furthermore, plasma cells/plasmablasts were significantly increased in both *C9orf72*^*−/−*^ and *Grn*^*−/−*^*;C9orf72*^*−/−*^ mice compared to *wild type* and *Grn*^*−/−*^ mice (Fig. 1d).

To characterize the temporal expansion of these aberrant B cell populations, we used multiparameter intracellular flow cytometry to characterize the B cell subsets in paired peripheral blood and splenocytes (**Extended Data Fig. 2g**, **Supplementary Table 2**). This approach revealed an age-dependent expansion of ABCs and activated B cells in spleen and peripheral blood in *Grn*^*−/−*^*;C9orf72*^*−/−*^ mice as early as 7 months compared to other genotypes (Fig. 1e–f). Finally, to determine whether the expansion of ABCs and activated B cells was due to clonal expansion, we analyzed the B cell receptor (BCR) repertoire in MZ B cells, ABCs, activated B cells, and plasma cells. While *wild type*, *Grn*^*−/−*^, and *C9orf72*^*−/−*^ mice maintained BCR diversity in various B cell subsets, clonal restriction as well as expansion of a few selective clones were evident in ABCs, activated B cells, and plasma cells in *Grn*^*−/−*^*;C9orf72*^*−/−*^ mice (Fig. 1g). Collectively, these results highlighted aberrant clonal expansion of B cell subsets as a major contributor to the immune defects in *Grn*^*−/−*^*;C9orf72*^*−/−*^ mice.

### TLR7 activation in *Grn*^*−/−*^*;C9orf72*^*−/−*^ B cells

To investigate the mechanisms leading to the abnormal B cell phenotype in *Grn*^*−/−*^*;C9orf72*^*−/−*^ mice, we performed a B cell-focused Gene Set Enrichment Analyses (GSEA) of differentially expressed genes (DEGs) using the CITE-seq dataset. This revealed that activated B cells in *Grn*^*−/−*^*;C9orf72*^*−/−*^ mice were enriched in genes related to endocytosis, leukocyte-mediated cytotoxicity, phagosome, and antigen processing/presentation pathways (Fig. 2a, **Extended Data Fig. 3a-b, Supplementary Table 2**). In addition, genes related to chemokine signaling, toll-like receptor (TLR) signaling, BCR signaling, and TNF signaling were increased in *C9orf72*^*−/−*^ and *Grn*^*−/−*^*;C9orf72*^*−/−*^ activated B cells compared to other genotypes (Fig. 2a, **Extended Data Fig. 3a-b**). Similar to activated B cells, *Grn*^*−/−*^*;C9orf72*^*−/−*^ ABCs and plasma cells/plasmablasts showed an enrichment of genes related to antigen processing and presentation. Additionally, TLR signaling, BCR signaling, lysosome, and phagosome pathways were upregulated in *C9orf72*^*−/−*^ and *Grn*^*−/−*^*;C9orf72*^*−/−*^ mice compared to *wild type* (**Extended Data Fig. 3a-b**). These results confirmed a profound B cell activation state and more importantly, raised the hypothesis that these signaling pathways might promote the aberrant expansion of ABCs and activated B cells in *Grn*^*−/−*^*;C9orf72*^*−/−*^ mice.

To determine how these dysregulated signaling pathways promote the expansion and differentiation of activated B cells and ABCs, we isolated splenic B cells from 4-month-old *wild type*, *C9orf72*^*−/−*^, *Grn*^*−/−*^, and *Grn*^*−/−*^*;C9orf72*^*−/−*^ mice before the significant expansion of ABCs and activated B cells (Fig. 1d). We assessed and compared a panel of B cell stimulation and co-stimulation pathways *in vitro* by treating these splenic B cells with an antibody cocktail against CD40, IgM, and CD19 to ligate and activate BCRs, 2′3′-cGAMP (2′3′-cyclic guanosine monophosphate-adenosine monophosphate) to activate STING pathway, R848 (Resiquimod) to activate TLR7 signaling, or CpG oligonucleotides 2395 (CPG2395) to activate TLR9 signaling. Next, we used flow cytometry, 12-plex cytokine assays, and bulk RNA-seq to characterize the responses of isolated B cells to these stimuli (Fig. 2b). Using flow cytometry, we showed that BCR, TLR7, or TLR9 stimulation led to a significant expansion of CD80/CD86^hi^;MHCII^hi^ B cells in *Grn*^*−/−*^*;C9orf72*^*−/−*^ splenocytes compared to *wild type*, *C9orf72*^*−/−*^, or *Grn*^*−/−*^ cells. By measuring cytokines released from stimulated B cells, we further showed that activating BCR signaling led to significant TNFα secretion in *Grn*^*−/−*^*;C9orf72*^*−/−*^ B cells, and imparted modest effects on IL-6 and IL-10 secretion (Fig. 2c–f). Likewise, TLR7 agonism led to drastic and consistent increases in TNFα, IL-6, and IL-10 secretion. In contrast, TLR9 stimulation did not increase TNFα or IL-6 secretion and only modestly increased IL-10 secretion (Fig. 2c–f). These data identified cell-intrinsic defects in *Grn*^*−/−*^*;C9orf72*^*−/−*^ B cells with functionally dysregulated BCR and TLR7 pathways.

Finally, we performed bulk RNA-seq to evaluate the transcriptomic changes in *wild type*, *C9orf72*^*−/−*^, *Grn*^*−/−*^, and *Grn*^*−/−*^*;C9orf72*^*−/−*^ splenic B cells after the stimulation of BCR, STING, TLR7 or TLR9 pathways. UMAP demonstrated that splenic B cells exhibited genotype-specific transcriptomic profile changes after BCR or TLR7 stimulation (Fig. 2g, **Extended Data Fig. 3c**, **Spplementary Table 3**). Indeed, TLR7 activation resulted in upregulation of genes related to FcγR-mediated phagocytosis and endocytosis in *Grn*^*−/−*^*;C9orf72*^*−/−*^ B cells compared to other genotypes (Fig. 2h). Similarly, BCR stimulation led to upregulation of genes related to actin cytoskeleton and BCR signaling pathways in *Grn*^*−/−*^*;C9orf72*^*−/−*^ B cells (**Extended Data Fig. 3c**). In contrast, TLR9 stimulation led to an increase in apoptosis, NFκB signaling, and TNF signaling pathways in *Grn*^*−/−*^*;C9orf72*^*−/−*^ B cells compared to other genotypes (**Extended Data Fig. 3d**). Although altered STING pathways have been previously reported in *C9orf72*^*−/−*^ dendritic cells^18^, 2′3′-cGAMP treatment in B cells showed minimal effects on cellular activation and cytokine secretion (Fig. 2c–d) and only modest changes in retinoic acid-inducible gene I (RIG-I) receptor and cytosolic DNA-sensing pathways in *Grn*^*−/−*^ B cells (**Extended Data Fig 3e**). These data indicate that impact of *Grn* and/or *C9orf72* on B cells may be mechanistically different than their effects on dendritic cells. Taken together, these *in vitro* results support the *ex vivo* CITE-seq data that aberrant activation of BCR and TLR7 signaling act as drivers of the expansion of B cell subsets in *Grn*^*−/−*^*;C9orf72*^*−/−*^ splenocytes.

### *Grn*^−/−^*;C9orf72*^−/−^ B cells produce anti-Robo3 autoantibodies

The age-dependent expansion of ABCs and activated B cells in 7- to 12-month-old *Grn*^*−/−*^*;C9orf72*^*−/−*^ mice is highly significant because both B cell subtypes have been associated with immune dysfunction in advanced aging and in autoimmune diseases, such as systemic lupus erythematosus (SLE)^21–25^. The aberrant expansion of both B cell subtypes in *Grn*^*−/−*^*;C9orf72*^*−/−*^ mice at young ages broached the hypothesis that these two B cell subtypes might produce CNS-targeting autoantibodies to promote neuroinflammation and neurodegeneration during brain aging. To test this, we used sera from 12-month-old *wild type*, *C9orf72*^*−/−*^, *Grn*^*−/−*^, and *Grn*^*−/−*^*;C9orf72*^*−/−*^ mice to perform Phage Immunoprecipitation Sequencing (PhIP-seq) (**Supplementary Table 4**), which is a high throughput assay for analyzing antibody-repertoire binding specificities^26,27^. To circumvent the limitations of PhIP-seq to detect conformational protein epitopes in 3D, we also used sera from 12-month-old *wild type*, *C9orf72*^*−/−*^, *Grn*^*−/−*^, and *Grn*^*−/−*^*;C9orf72*^*−/−*^ mice to screen Human Proteome Microarrays (HuProt^™^, CDI Labs), which covered >15,000 GST-tagged human proteins (**Supplementary Table 5**). As expected, sera from *wild type* mice did not contain significant reactivity to autoantigens, whereas sera from *C9orf72*^*−/−*^, *Grn*^*−/−*^, and *Grn*^*−/−*^*;C9orf72*^*−/−*^ mice each contained autoantibodies that recognized multiple genotype-specific antigens (Fig. 3a, **Supplementary Tables 4–5**). By combining the results from PhIP-seq and the Proteome Microarray, we further narrowed down to 70 shared antigens including 46 targeted by autoantibodies from *C9orf72*^*−/−*^ mice, 44 from *Grn*^*−/−*^ mice, and 42 from *Grn*^*−/−*^*;C9orf72*^*−/−*^ mice (Fig. 3b, **Supplementary Table 6**).

Using databases from the Human Protein Atlas, we developed Sankey plots to classify these antigens based on their subcellular localizations, including nuclear, cytoplasmic, endoplasmic reticulum, plasma membrane, or multiple locations, and different cell types in the central nervous system (CNS), including inhibitory neurons, excitatory neurons, oligodendroglia, oligodendroglia precursor cells (OPCs), microglia, astrocytes, rod cells and cone cells (Fig. 3c). Among the 23 CNS antigens, we focused on Robo3 (roundabout guidance receptor 3) because (1) Robo3 is a membrane-bound protein that has critical roles in guiding commissural axons during spinal cord development^28^, (2) in the adult nervous system, Robo3 proteins can be detected in synapses and astrocytic processes^29–31^, and (3) mutations in the human *ROBO3* gene cause hindbrain axon midline crossing defects, horizontal gaze palsy, and progressive scoliosis (HGPPS) syndrome^32^. From PhIP-seq datasets, we identified 6 peptides in the first Ig domain and 3 peptides in the first cytoplasmic domains in Robo3 that were recognized by autoantibodies (Fig. 3d). Furthermore, western blots using recombinant Robo3 proteins and sera from *wild type*, *C9orf72*^*−/−*^, *Grn*^*−/−*^, and *Grn*^*−/−*^*;C9orf72*^*−/−*^ mice confirmed the presence of these autoantibodies from *C9orf72*^*−/−*^, *Grn*^*−/−*^, and *Grn*^*−/−*^*;C9orf72*^*−/−*^ mice, but not in sera from *wild type* mice (Fig. 3e). To further understand the kinetics of the rise in anti-Robo3 autoantibodies, we developed ELISA assays using recombinant Robo3 proteins and showed that anti-Robo3 autoantibodies can be detected in *C9orf72*^*−/−*^, *Grn*^*−/−*^, and *Grn*^*−/−*^*;C9orf72*^*−/−*^ mice from 4, 7 and 12-month-old groups (Fig. 3f). Finally, we used confocal microscopy to show that the expression of Robo3 proteins was low in thalamic neurons in 12-month-old *wild type*, *C9orf72*^*−/−*^ and *Grn*^*−/−*^ mouse brains. In contrast, *Grn*^*−/−*^*;C9orf72*^*−/−*^ mouse brain showed a robust increase of Robo3 in both astrocytes and neurons (Fig. 3g).

To connect these results with human disease, we examined the presence of anti-Robo3 autoantibodies using CSF samples from *C9orf72* or *GRN* mutation carriers at presymptomatic or symptomatic stages and non-carrier controls (**Extended Data Fig. 4a**, **Supplementary Table 7**). These results showed that, as a group, symptomatic *C9orf72* carriers had significantly higher anti-Robo3 autoantibody titers in their CSF samples than non-carrier controls, whereas *GRN* carriers and presymptomatic *C9orf72* carriers showed no significant increases (**Extended Data Fig. 4b-c**). Interestingly, anti-Robo3 titers in presymptomatic and symptomatic *GRN* carriers positively correlated with complement C1q and C3b (Fig. 3h). Given the well-established roles of C1q and C3b in *Grn* mouse models and in FTLD-GRN patients^13,14,33^, these results suggest that a loss of tolerance and the exacerbation of adaptive immune responses leading to the production of CNS-targeting autoantibodies, such as anti-Robo3, may promote neuroinflammation in disease progression in *C9orf72* and *GRN* mutation carriers.

### Loss of *Grn* and *C9orf72* aggravated astrogliosis

To determine the impact of autoimmunity to astrocytes in *Grn*^*−/−*^*;C9orf72*^*−/−*^ mice, we examined brain pathology in 7- and 12-months-old *wild type*, *C9orf72*^*−/−*^, *Grn*^*−/−*^, and *Grn*^*−/−*^*;C9orf72*^*−/−*^ mice and found no expansion of GFAP^+^ astrocytes in *C9orf72*^*−/−*^ mice compared to *wild type* mice (Fig. 4a–b). Interestingly, *Grn*^*−/−*^ mice showed modest astrogliosis in the ventral thalamus at 7-month-old, which became more pronounced by 12-month-old^13,14,34^. In contrast, *Grn*^*−/−*^*;C9orf72*^*−/−*^ mice exhibited even more prominent thalamic astrogliosis at 7- and 12-months-old (Fig. 4a–b). By 12-month-old, the astrogliosis phenotype in *Grn*^*−/−*^*;C9orf72*^*−/−*^ mice extended beyond the ventral thalamus, and affected amygdala and piriform cortex, where the density of GFAP^+^ astrocytes nearly doubled or tripled in these regions (Fig. 4a, 4c). To further characterize the gliosis phenotype, we performed bulk RNA-seq using hemi-brains from 7- and 12-month-old *wild type*, *C9orf72*^*−/−*^, *Grn*^*−/−*^, and *Grn*^*−/−*^*;C9orf72*^*−/−*^ mice (**Extended Data Fig. 5a-c**). Bioinformatics analyses revealed age-dependent increases in genes related to BCR signaling, phagosomes, TLR signaling, complements, and lysosomes in the *Grn*^*−/−*^*;C9orf72*^*−/−*^ mouse brain (**Extended Data Fig. 5d-g, Supplementary Table 8**). Furthermore, gene network interaction analysis in the CNS revealed significantly enriched interactions between *Tlr7* and genes implicated in phagosomes, endolysosomal trafficking, and B cell activation, including *Rab5c*, *Rab7b*, *Lamp1*, *Ctss*, *Fcgr2b*, and *Cd86* (**Extended Data Fig. 5h**).

The upregulation of the Fc receptor, *Fcgr2b,* and the costimulatory molecule, *Cd86,* raised the hypothesis that the expansion of activated B cells leads to increased immunoglobulin-mediated endocytosis, autoantigen processing/presentation, and autoantibody production, which might promote the neuroinflammation phenotypes in the *Grn*^*−/−*^*;C9orf72*^*−/−*^ mouse brain. To test this, we first examined the integrity of the blood-brain barrier (BBB) in 12-month-old *wild type*, *C9orf72*^*−/−*^, *Grn*^*−/−*^, and *Grn*^*−/−*^*;C9orf72*^*−/−*^ mice by intravenous injection of 40 kDa FITC-conjugated Dextran. In 12-month-old *wild type* mouse brains, essentially all the dextran dye was retained inside blood vessels without leakage into the brain parenchyma, whereas in *C9orf72*^*−/−*^ and *Grn*^*−/−*^ mouse brain a small amount of dextran dye was detected in microglia or astrocytes (Fig. 4d, top row). In contrast, most of the Dextran dye was engulfed by Iba1^+^ microglia and GFAP^+^ astrocytes in *Grn*^*−/−*^*;C9orf72*^*−/−*^ mouse brain (Fig. 4d, upper row). Furthermore, *Grn*^*−/−*^*;C9orf72*^*−/−*^ mouse brain exhibited extensive deposits of IgG in Iba1^+^ microglia and GFAP^+^ astrocytes (Fig. 4d, lower row). Consistent with these results, immunogold electron microscopy (IEM) confirmed that significantly more IgG^+^ immunogold particles were detected outside the basement membrane of brain endothelial cells in *Grn*^*−/−*^*;C9orf72*^*−/−*^ thalami (Fig. 4e–f).

To further investigate how loss of PGRN and C9orf72 impact astroglial phenotypes, we performed single-nuclei RNA-seq (snRNA-seq) using microdissected thalami from 12-month-old *wild type*, *C9orf72*^*−/−*^, *Grn*^*−/−*^, and *Grn*^*−/−*^*;C9orf72*^*−/−*^ mice. In total, we analyzed 23 samples, including 3–4 biological replicates per genotype (**Extended Data Fig. 6a-e**, **Supplementary Table 9**). On average, we obtained 3,767 single nuclei per sample with 5,695 unique molecular identifiers (UMIs) and 2,270 genes per nucleus (**Supplementary Table 9**). Unsupervised clustering and visualization via UMAP revealed 16 unique clusters including five excitatory neuron clusters (c1, c2, c3, c4, and c5), two inhibitory neuron clusters (c8, c9), oligodendroglia (c10), immature oligodendroglia cluster (c11), microglia (c12), astrocytes (c13), endothelial cells (c14), pericytes (15), and fibroblasts (c16) (Fig. 4g, **Extended Data Fig. 6f-g**). Consistent with the glial pathology, snRNA-seq showed an expansion in the astrocyte cluster (c13) in *Grn*^*−/−*^*;C9orf72*^*−/−*^ thalami. Subclustering analyses of the astrocyte cluster revealed a reduction in subcluster 2, which expressed homeostatic astrocyte genes, *Gria2*, *Slc7a10*, and *Slc1a3*, and an expansion of subcluster 4, which expressed reactive astrocyte genes, *Gfap*, *C4b*, *Apoe*, and *Aqp4*, in *Grn*^*−/−*^*;C9orf72*^*−/−*^ astrocytes (Fig. 4h–i, **Extended Data Fig. 7a-g**). GSEA using Gene Ontology (GO) and Kyoto Encyclopedia of Genes and Genomes (KEGG) databases revealed that the DEGs in *C9orf72*^*−/−*^ astrocytes were enriched in pathways related to Rho protein signal transduction, regulation of inflammatory response, lipid biosynthetic process, and fatty acid metabolism, while genes related to phagosome function were downregulated; by comparison, *Grn*^*−/−*^ astrocytes showed increased expression in genes related to receptor-mediated endocytosis, humoral immune response, lysosome, and ferroptosis. In contrast, *Grn*^*−/−*^*;C9orf72*^*−/−*^ astrocytes showed increased expression in genes identified in both *C9orf72*^*−/−*^ astrocytes and *Grn*^*−/−*^ astrocytes (Fig. 4j–l, **Extended Data Fig. 7h-i**, **Supplementary Table 10**). Consistent with these findings, a heatmap of DEG-defined GO terms and KEGG pathways in astrocytes confirmed these results and revealed additional transcriptomic upregulation in *Grn*^*−/−*^*;C9orf72*^*−/−*^ astrocytes related to inflammatory response and antigen processing and presentation pathways. In addition, targeted pathway analyses in *wild type*, *C9orf72*^*−/−*^, *Grn*^*−/−*^, and *Grn*^*−/−*^*;C9orf72*^*−/−*^ astrocytes showed greater downregulation of synaptic functions, and upregulation of lipid biosynthetic pathways, angiogenesis, and BCR signaling in *Grn*^*−/−*^*;C9orf72*^*−/−*^ astrocytes. Together, these results demonstrated that loss of *Grn* and *C9orf72* synergistically aggravated the transcriptomic profiles of *Grn*^*−/−*^*;C9orf72*^*−/−*^ astrocytes.

### TLR7 inhibition mitigates autoimmunity and astrogliosis in *Grn*^*−/−*^*;C9orf72*^*−/−*^ mice

The upregulation of Robo3 in *Grn*^*−/−*^*;C9orf72*^*−/−*^ astrocytes suggested that the anti-Robo3 autoantibodies produced by ABCs and activated B cells may more readily cross the BBB and exacerbate astrogliosis in *Grn*^*−/−*^*;C9orf72*^*−/−*^ mice. If so, then active immunization with Robo3 extracellular domain (ECD) should lead to more prominent astrogliosis in young *Grn*^*−/−*^*;C9orf72*^*−/−*^ mice. To test this, we developed a 10-week protocol to immunize 4-month-old *wild type*, *C9orf72*^*−/−*^, *Grn*^*−/−*^, and *Grn*^*−/−*^*;C9orf72*^*−/−*^ mice with the Robo3 ECD. Immunizations were initiated on week 0 in mice with Robo3 ECD or PBS (vehicle) prepared with complete Freund’s adjuvant (CFA) and followed with biweekly boosts of Robo3 ECD or vehicle mixed with incomplete Freund’s adjuvant (IFA). On week 10, at age 6.5 months, these mice were euthanized for brain tissue collection (Fig. 5a). Histology results showed that neither vehicle nor Robo3 ECD immunization affected GFAP^+^ astroglial density in the ventral thalamus in *wild type* and *C9orf72*^*−/−*^ mice (Fig. 5b–c). While *Grn*^*−/−*^ mouse brains showed a modest increase in GFAP^+^ astroglial density in the ventral thalamus, there was no difference in *Grn*^*−/−*^ mice immunized with vehicle or Robo3 ECD (Fig. 5b–c). In contrast, *Grn*^*−/−*^*;C9orf72*^*−/−*^ mice immunized with Robo3 ECD showed a significant increase in GFAP^+^ astroglial density, whereas those that received vehicle treatment showed modest astrogliosis like that in *Grn*^*−/−*^ mice (Fig. 5b–c). In contrast, Robo3 ECD immunization did not affect the density of Iba1^+^ microglia in 6.5-month-old *wild type*, *C9orf72*^*−/−*^, *Grn*^*−/−*^, and *Grn*^*−/−*^*;C9orf72*^*−/−*^ mice (**Extended Data Fig. 8a-b**). Overall, these results supported that immunization of a single CNS antigen, Robo3 ECD, is sufficient to promote astrogliosis phenotypes in young *Grn*^*−/−*^*;C9orf72*^*−/−*^ brains.

Conversely, since clonally expanded ABC and activated B cells exhibited pronounced activation of the TLR7 pathway, we asked whether pharmacological inhibition of TLR7 could mitigate ABC and activated B cell expansion and reduce anti-Robo3 autoantibodies to alleviate astrogliosis phenotypes in older *Grn*^*−/−*^*;C9orf72*^*−/−*^ mice. To test this, we used oral gavage to deliver the TLR7 inhibitor M5049 (Enpatoran, 1 mg/kg)^35^ or placebo daily to 8-month-old *wild type*, *C9orf72*^*−/−*^, *Grn*^*−/−*^, and *Grn*^*−/−*^*;C9orf72*^*−/−*^ mice (Fig. 5a). After 8 weeks, these mice were euthanized and their plasma, splenocytes and brains were collected. In the periphery, M5049 significantly diminished the percentage of Tbet^+^;CD11c^+^ ABC and CD80/86^hi^;MHCII^hi^ activated B cells in splenocytes as assessed by flow cytometry (Fig. 5e–f). Further, M5049 reduced anti-Robo3 plasma titers in *Grn*^*−/−*^*;C9orf72*^*−/−*^ mice (Fig. 5d). In the CNS, our results showed that this treatment regimen can achieve 5–10 ng/g brain weight of M5049 concentration in mouse brains (**Extended Data Fig. 8c**). Immunohistochemistry showed that M5049 treatment significantly reduced GFAP^+^ astroglial density in the ventral thalamus of 10-month-old *Grn*^*−/−*^*;C9orf72*^*−/−*^ mice, but not in *wild type*, *C9orf72*^*−/−*^, or *Grn*^*−/−*^ mice (Fig. 5g–h). Interestingly, the same treatment also reduced the density of Iba1^+^ microglia in *Grn*^*−/−*^ and *Grn*^*−/−*^*;C9orf72*^*−/−*^ mouse brain, but not in *wild type* or *C9orf72*^*−/−*^ mouse brain (Fig. 5i, **Extended Data Fig. 8d**). Thus, systemic targeting of TLR7 *in vivo* significantly ameliorated ABC expansion and dampened astrogliosis specifically in *Grn*^*−/−*^*;C9orf72*^*−/−*^ mice.

To determine the functional outcome of M5049 treatment on astroglial activation, we used confocal microscopy to examine the IgG engulfment by astrocytes and microglia in *wild type*, *C9orf72*^*−/−*^, *Grn*^*−/−*^ and *Grn*^*−/−*^*;C9orf72*^*−/−*^ mice. These results showed that M5049 treatment significantly reduced the ability of *Grn*^*−/−*^*;C9orf72*^*−/−*^ astrocytes and microglia to engulf IgG (Fig. 5j–m). Importantly, M5049 treatment partially restored the synaptic loss phenotypes in *Grn*^*−/−*^ and *Grn*^*−/−*^*;C9orf72*^*−/−*^ mouse brain (Fig. 5n–o). Given the elevated expression of genes related to endocytosis and phagosomes in *Grn*^*−/−*^ and *Grn*^*−/−*^*;C9orf72*^*−/−*^ astrocytes, we also examined whether TLR7 can cell autonomously affect FcR- and TLR2-mediated phagocytosis in *wild type*, *C9orf72*^*−/−*^, *Grn*^*−/−*^ and *Grn*^*−/−*^*;C9orf72*^*−/−*^ astrocytes. To this end, we prepared primary astrocyte cultures from neonatal *wild type*, *C9orf72*^*−/−*^, *Grn*^*−/−*^, and *Grn*^*−/−*^*;C9orf72*^*−/−*^ mouse cortices as previously described^13,14,36^, and used FITC-conjugated IgG and pHrodo^™^ Green Zymosan to assess Fc receptor (FcR)-mediated and TLR2-mediated phagocytosis, respectively (**Extended Data Fig. 9a**). The phagocytosis assays were conducted in basal media (unstimulated) or treated with TLR7 agonist R848, TLR9 agonist CPG2395, or STING agonist cGAMP. Our results showed that both *wild type* and *C9orf72*^*−/−*^ astrocytes exhibited modest FcR-mediated phagocytic activity that was further increased by TLR7 agonist R848, whereas CPG2395 and 2′3′-cGAMP showed no effects (**Extended Data Fig. 9b**). Compared to *wild type* and *C9orf72*^*−/−*^ astrocytes, *Grn*^*−/−*^ and *Grn*^*−/−*^*;C9orf72*^*−/−*^ astrocytes exhibited significantly higher FcR-mediated phagocytic activity, which could be further enhanced by TLR7 agonism, but not affected by TLR9 or STING activation (**Extended Data Fig. 9b**). In contrast, TLR2-mediated phagocytic activities in *wild type*, *C9orf72*^*−/−*^, *Grn*^*−/−*^ and *Grn*^*−/−*^*;C9orf72*^*−/−*^ astrocytes were quite low and the TLR7 agonist R848 had no or modest effects in promoting this activity (**Extended Data Fig. 9c**). These data support that TLR7 inhibitor treatment could impact not only the periphery, but also the CNS to diminish astrogliosis and IgG-mediated astrocyte phagocytic activity which preserved synaptic density in *Grn*^*−/−*^*;C9orf72*^*−/−*^ mouse brains.

## DISCUSSION

Case-controlled studies^37,38^, Genome-Wide Association Studies^39^, and single-cell transcriptomics of postmortem brain tissues^40,41^ support that the pathogenesis of FTD involves intricate interactions between systemic autoimmunity and neuroinflammation. However, it remains unclear how dysfunction in adaptive immunity contributes to neuroinflammation and neurodegeneration in FTD. Here we show that concurrent loss of *Grn* and *C9orf72* leads to a breakdown of immune tolerance and severe autoimmune dysfunction characterized by clonal expansion of ABCs, activated B cells and plasma cells. Furthermore, *Grn* and *C9orf72* deficiencies led to the production of autoantibodies targeting multiple CNS antigens, including astroglial antigen Robo3, to promote astroglial pathology. Single-cell transcriptomic profiling of astrocytes in *Grn*^*−/−*^*;C9orf72*^*−/−*^ mouse brain revealed robust features of the inflammatory response, immune effector process, and antigen processing and presentation. Interestingly, immunization of Robo3 ECD in young *Grn*^*−/−*^*;C9orf72*^*−/−*^ mice accelerates the astrogliosis phenotype, whereas treatment with the TLR7 inhibitor M5049 rescued B cell defects, reduced anti-Robo3 autoantibodies, and mitigated astrogliosis and synaptic loss in *Grn*^*−/−*^*;C9orf72*^*−/−*^ mice. Collectively, these results provide critical mechanistic insights into how TLR7-mediated autoimmune dysfunction contributes to neurodegeneration by targeting Robo3 to drive neuroinflammation and neurodegeneration in FTD.

Originally identified as an atypical B cell subset that age-dependently accretes in mice and are enriched in autoimmune-prone mouse strains^21,22^, age-associated B cells (ABC) are recognized for their unique transcriptomic signatures and activation requirements. A key feature of ABCs is their expression of the Th1 transcription factor T-bet, which, upon overexpression, is sufficient to induce the acquisition of the ABC phenotype^42^. ABCs are also often identified by myeloid cell markers, such as CD11c^24,43^. In addition, engagement of the innate endosomal immunoreceptor, TLR7, together with BCR ligation, and IFNγ promotes the generation, differentiation, and expansion of ABCs^43,44^. Since their discovery, ABCs have been implicated in autoimmunity and antiviral immune responses^42,45–47^. In both scenarios, ABCs produce high titers of autoantibodies that further contribute to autoimmunity and efficient viral clearance. Our CITE-seq data identify clonal restriction and oligoclonal expansion of ABCs and activated B cells as a major cause of autoimmune dysfunction in *Grn*^*−/−*^*;C9orf72*^*−/−*^ mice. Together, ABCs and activated B cells account for ~20% of total B cells in the splenocytes in *Grn*^*−/−*^*;C9orf72*^*−/−*^ mice, which can be detected in the peripheral blood of *Grn*^*−/−*^*;C9orf72*^*−/−*^ mice in an age-dependent manner. In support of their contributions to autoimmunity, ABCs and activated B cells in *Grn*^*−/−*^*;C9orf72*^*−/−*^ mice produce antibodies that target a broad repertoire of CNS antigens. Interestingly, anti-Robo3 antibodies can be detected in the CSF of human *C9orf72* or *GRN* mutation carriers, and their titers positively correlate with complement C1q and C3b, which promote neuroinflammatory phenotypes in *Grn* mouse models and in FTLD patients^13,14,33,48^. These results suggest that anti-Robo3 autoantibodies and perhaps other CNS-targeting autoantibodies, produced by autoimmune dysfunction, may have similar impact on glial pathology and synaptic degeneration in patients with *C9orf72* and/or *GRN* mutations.

Mechanistically, the aberrant activation of TLR7 signaling in *Grn*^*−/−*^*;C9orf72*^*−/−*^ B cells is likely caused by defects in endolysosomal trafficking. Indeed, C9orf72 has critical roles in the autophagy-lysosome pathway^16,49–51^, whereas PGRN regulates the transition from late endosomes to early lysosomes and the biogenesis of mature lysosomes^12,52^. It is conceivable that concurrent loss of PGRN and C9orf72 synergizes the intracellular trafficking defects of TLR7, rendering it more prone to become activated under steady state and upon stimulation by a TLR7 agonist. In support of these results, the TLR7/8 antagonist M5049 (Enpatoran) effectively reduced ABC and activated B cells and mitigate astrogliosis-related phenotypes in *Grn*^*−/−*^*;C9orf72*^*−/−*^ mice linking TLR7 to FTD. Given the clinical implications of M5049 in treating SLE^53–56^, it is conceivable that inhibiting TLR7 may be beneficial in mitigating autoimmunity, neuroinflammation, and neurodegeneration in FTD. Future studies will be required to determine the roles of B cells and TLR7 in the treatment for FTD.

## METHODS

### Animals

All experiments were conducted in accordance with the University of California San Francisco, Washington University, and San Francisco VA Healthcare System Institutional Animal Care and Use Committee guidelines that followed the National Institute of Health guidelines (IACUC Protocol #AN169548, ACORP 24–019). Mice carrying deletions of exons 2–13 of the mouse *Grn* gene were used from previous studies in our laboratory^57^ and is available at the Jackson Laboratory (B6.129S4(FVB)-*Grn*^*tm1.1Far*^/Mmjax, #021210, RRID:MMRRC_036771-JAX). *C9orf72*^*−/−*^ mice were obtained from Jackson Laboratory (C57BL/6J-*C9orf72e*^*m5Lutzy*^/J, #027068, RRID:IMSR_JAX:027068) with the F12 *C9orf72* null allele (deletion of 79bp in exon 2) as previously described^58^. *Grn*^*−/−*^*;C9orf72*^*−/−*^ double knockout mice were generated by intercrossing *Grn*^*−/−*^ and *C9orf72*^*−/−*^ and between the compound heterozygote mice.

### Flow cytometry

Splenocytes and whole blood was treated with 5x volume of ACK Lysis Buffer (Gibco) for 5 minutes at room temperature to lyse red blood cells. Following lysis, cells were washed with PBS and resuspended in Cell Staining Buffer (BioLegend). LIVE/DEAD stain (0.5 μL per 1×10^6^ cells; Biolegend) was added to cells and incubated for ≥20 minutes at 4°C, followed by CD16/CD32 block (BioLegend) for ≥15 minutes. Cells were stained with fluorochrome-conjugated antibodies for surface markers for ≥30 minutes at 4°C, washed, and fixed in 2% paraformaldehyde for 30 minutes at room temperature. For intracellular staining, cells were washed with Intracellular Permeabilization Buffer or Intranuclear Perm Buffer (BioLegend), stained with intracellular/intranuclear antibodies, and incubated for 45 minutes at room temperature. After the final washes, cells were resuspended in Cell Staining Buffer and analyzed using a BD Biosciences FACS Fusion at the San Francisco VA Healthcare System Flow Cytometry Core Facility or at Oklahoma Medical Research Foundation (OMRF). All steps were conducted in the dark and at 4°C where applicable. All flow cytometric data acquisitions were done with FACS Diva software v.7 (BD) and analyzed with FlowJo v10.9.0 (LLC).

### Primary astrocyte cultures and phagocytosis assays

Primary astrocyte cultures were prepared as previously described^14,36^. Mixed glial cultures were prepared in T-75 flasks coated with 5 mL of poly-L-lysine (PLL) for 30 minutes to overnight. After washing with DPBS, flasks were filled with 7 mL Growth Media and incubated at 37°C. Postnatal day 3–5 (P3-P5) pups were anesthetized on ice, decapitated, and the cerebral cortices were extracted by removing the skull and meninges using forceps. The brains were placed in cold HBSS, and the cortex was carefully dissected. Cortical tissue was chopped into small pieces and transferred to 15 mL conical tubes with 3 mL HBSS. After dissection, the chopped cortex was transferred to fresh tubes with 3 mL of Growth Media (with GM-CSF) and subsequently added to T-75 flasks containing 7 mL Growth Media, for a total of 10 mL. Flasks were incubated at 37°C for 3 days, with media changes performed on day 3. A 96-well plate was coated with 70 μL of poly-L-lysine for 30 minutes to overnight, depending on prior usage. After washing with PBS (Ca^2+^/Mg^2+^-free), the last wash was retained until cell seeding. Primary astrocytes were harvested by shaking off microglia for 3 hours at 200 rpm and 37°C at DIV 10–14. Astrocytes were seeded in 96-well or 24-well plates. Stimulation was performed with R848 (Resiquimod, InvivoGen, Cat# tlrl-r848, concentration used: 1 μg/ml), 2′3′-cGAMP (InvivoGen, Cat# tlrl-nacga23–02, concentration used: 5 μg/ml), or CpG2395 (InvivoGen, Cat# tlrl-2395–1, concentration used: 0.2 μM, 2 μL for 96-well, 5 μL for 24-well). After a 3-hour incubation at 37°C, the media was removed, and cells were labeled with CellTracker CMTPX Red dye (1 μL of 1 mM solution in 1 mL serum-free DMEM) for 30 minutes, followed by washing with DMEM. Cells were then treated with phagocytic agents (e.g., IgG-FITC Latex Beads or Zymosan-Green). The plate was placed in an IncuCyte^®^ Live-Cell Analysis System with phase contrast and fluorescence channels to monitor phagocytosis for 10 hours. Overlapping phagocytosis and apoptosis signals with cytoplasmic labeling per total cell count based on 2x images was used to normalize the phagocytotic count and apoptotic rate within each technical replicate. A total of 4 technical replicates per each biological replicate were used to calculate the mean and standard deviation.

### Antibody usages

#### Flow cytometry:

Anti-CD93-APC (Invitrogen, 17-5892-82, clone: AA4.1, 1:100), anti-CD23-BV786 (BD Biosciences, 563988, clone: B3B4, 1:100), anti-CD86-PE/CF594 (BD Biosciences, 567592, clone: GL1, 1:100), anti-CD80-PE/CF594 (BD Biosciences, 562504, clone: 16–10A1, 1:100), anti-CD138-BV421 (BD Biosciences, 562610, clone: 281–2, 1:100), anti-IgD-BV650 (BioLegend, 405721, clone: 11–26c.2a, 1:100), anti-IgM-PE/Cy7 (Invitrogen, 14-5790-82, clone: 11/41, 1:100), anti-CD11c-FITC (Invitrogen, 11-0114-82, clone: N418, 1:100), anti-CD5-BV605 (BD Biosciences, 563194, clone: 53.7.3, 1:100), anti-MHC II-PE/Cy5 (Invitrogen, 56-5321-82, clone: M5/114.15.2, 1:100), LIVE/DEAD staining (Cytek, 13–0865-T500, 1:200), anti-CD11b (BioLegend, 101211, clone: M1/70, 1:100), anti-Tbet-PE (BioLegend, 644809, clone: 4B10, 1:100), anti-CD19-BV711 (BD Biosciences, 563157, clone: 1D3, 1:100).

#### Immunohistochemistry:

##### Primary antibodies.

The primary antibodies used include IBA1 (1:3000; 019–19741, Wako), GFAP (1:3000, Z0334, Dako), CD68 (1:1000, ab125212, Abcam), Fcer1a (1:300, 14-5898-82, Invitrogen), FABP5 (1:2000, AF1476-SP, R&D Systems), NPC2 (1:1000, PA5–51463, Proteintech), Lipidtox (1:300, H34476, Invitrogen), APOE (1:200, ab1906, Abcam), Coro1c (1:5000, 14749–1-AP, Proteintech), S100β (1:1000, ab11178, Abcam). Anti-synaptophysin (1:1000, 101–004, Synaptic Systems), anti-PSD-95 (1:200, MA1–045, Invitrogen), anti-Robo3 (1:100, 20220–1-AP, Proteintech).

##### Secondary Antibodies.

The secondary antibodies used include goat anti-rabbit IgG antibody (H+L), biotinylated (1:300; BA-1000, Vector Laboratories), goat anti-chicken IgG antibody (H+L), biotinylated (1:300; BA-9010), goat anti-mouse IgG antibody (H+L), biotinylated (1:300; BA-9200, Vector Laboratories), Alexa Fluor 488 (1:300; A-11008, Invitrogen), Alexa Fluor 488 (1:300; A-11055, Invitrogen), Alexa Fluor 488 (1:300; A-21206, Invitrogen), Alexa Fluor 555 (1:300; A-31570, Invitrogen) Alexa Fluor 568 (A11004), Alexa Fluor 555 (1:300; A-31572, Invitrogen), Alexa Fluor 594 (1:300, A-11042, Invitrogen), Alexa Fluor 555 (1:300; A-21432, Invitrogen), Alexa Fluor 647 (1:300; A-31571, Invitrogen, Alexa Fluor 647 (1:300, A-21449, Invitrogen).

### Immunohistochemistry:

Immunohistochemical stains using 3’3 diaminobenzidine (DAB) were performed on 40-μm free-floating sections of 4% PFA-fixed mouse brains. The sections were rinsed in 1X Tris buffered saline (1X TBS) and then quenched with 10% methanol and 3% H_2_O_2_ in 1X TBS for 5 minutes. Sections were washed three times in 1X TBS and blocked for 0.5–1 hour with TBS+++ (10% goat serum, 3% bovine serum albumin (BSA), 1% glycine and 0.4% Triton X100). Primary antibodies were incubated overnight at room temperature. Biotinylated antibodies were incubated for 1 hour at room temperature and rinsed three times with 1X TBS. The VECTASTAIN Elite ABC HRP system with A (Avidin) and B (Biotinylated HRP) complexes (PK-6100) was used to amplify staining specificity. The signals were developed in DAB reaction solution (0.05% DAB and 0.05% H_2_O_2_ in 0.1M Tris, pH 8.0) and stopped with three washes in 0.1M Tris (pH 8.0). The tissue was counterstained with cresyl violet staining and dehydrated with 100% ethanol and Xylene before mounted on a slide. Slides were coverslipped using Cytoseal mounting medium. Images were captured using an Aperio AT2 scanner and Aperio ImageScope (Leica Biosystems). For stereology-based quantification, IBA1^+^ microglia and GFAP^+^ astrocytes were counted using optical fractionator-based unbiased method using Stereo Investigator on a PC that is attached to an Olympus BX5 microscope with a motorized XYZ stage (MBF Biosciences). Immunofluorescence stains were performed on 40-μm free-floating sections which were washed three times with 1X TBS and blocked in TBS+++ or SuperBlock T20 (ThermoScientific 37516) for 0.5–1 hour. The tissue was incubated overnight at room temperature. Alexa fluorophore conjugated secondary antibodies were diluted in TBS+++ (1:300) were added on the following day for 1h. The slides were then stained with DAPI and mounted on a slide before being coverslipped.

### Bulk RNA-seq: analysis and GSEA

Bulk RNA sequencing of left-brain hemispheres of 7 and 12-month-old mice was performed on Illumina HiSeq4000 machine (with an average depth of 35 million reads/sample) through UCSF Genomics Core Facility. For each sample, RNA was extracted using the QIAGEN RNeasy Mini kit following the manufacturer’s instructions and loaded as input for cDNA implication. RNA concentration was measured using Agilent 2100 Bioanalyzer with RIN >7, which was diluted to 10 ng/μL with the total RNA amount of 100 ng per sample provided to the Genomics Core Facility. Libraries were constructed by the Core using Takara SMART-seq (v4) and Illumina Nextera XT. Library pools were quality-controlled and normalized using Illumina MiniSeq before pair-ended (150-bp reads). Finally, reads were aligned to hg38 using STAR (v2.7.2.b). EdgeR (version 3.34.1) in R software was used to normalize raw counts and perform differential gene expression analyses. Raw reads were assessed for quality with FastQC and aligned to the GRCh38 reference genome with using STAR (v2.7.2.b)] using the Ensembl annotation; gene-level counts were summarized with featureCounts. Genes with low expression were removed using filterByExpr (edgeR), retaining genes with sufficient counts in the smallest group, and library composition was normalized by the trimmed mean of M-values (TMM) method. Counts were normalized and variance-stabilized (VST) using DESeq2. Differential expression was assessed in limma by fitting a linear model with group, age, and condition as the variables of interest, followed by empirical Bayes moderation of standard errors (lmFit, contrasts.fit, eBayes). Sample-level quality and batch structure were evaluated by principal component analysis and hierarchical clustering of the normalized expression matrix. P values were adjusted for multiple testing using the Benjamini–Hochberg false discovery rate (FDR), and genes with FDR < 0.05 and |log2 fold change| > 0.2 were considered differentially expressed. Pathway-level changes were evaluated by gene set enrichment analysis (GSEA) using the fgsea and clusterProfiler R packages with gene sets retrieved from canonical pathways (Reactome, KEGG, and GO) collections restricted to *Mus musculus*. Genes were ranked by the limma moderated by −log_10_(*p*) × sign(log_2_*FC*) across all tested genes without prior significance filtering, and enrichment was computed by the fast preranked algorithm with permutations and gene set size limits of to genes. Normalized enrichment scores (NES) were used to compare across gene sets, and enrichment was considered significant at an FDR-adjusted p value < 0.05. Gene regulatory networks were inferred from the normalized expression matrix using GENIE3, which decomposes network inference into per-target-gene random forest regressions and ranks candidate regulator–target links by variable importance. Input features were restricted to the candidate regulators. Random forests were grown with trees per target gene using the square-root feature-sampling scheme, with a fixed random seed for reproducibility. The resulting weighted adjacency matrix was converted to a ranked edge list, and the top weighted links (top 200) were retained to construct the final network.

### CITE-seq, Cell Hashing, and BCR sequencing

Four animals per genotype at 7 months of age were used, each genotype was equally split between male and female, and 2 independent experimental replicates were performed. Splenocytes were isolated by ficoll underlayment of spleen cell suspensions followed by a 20 min density-gradient centrifugation at 600*g* at room temperature, with no brake. 0.5×10^6^ splenocytes per sample were resuspended in 25ul cell staining buffer (Biolegend 420201) containing 2% TruStain FcX PLUS block (BioLegend 156603) for 10 min on ice. 25ul of reconstituted barcoded Total Seq C cell surface staining antibodies (TotalSeq C custom mouse panel of 138 monoclonal antibodies, BioLegend 900004599) were added to each tube and incubated for 20min on ice. A unique barcoded Cell Hashing antibody (0.5 ug/sample) targeting CD45 (clone 30/F11) and MHC class I H-2b (clone M1/42) was added to each tube and incubated 30min on ice. Cells were washed 2 times with 2ml resuspension buffer and counted by Countess (Invitrogen). Equivalent numbers of live cells per sample were combined into one pool. Cell sorting to remove Sytox blue^+^ debris was performed on a BD FACS Fusion at the San Francisco VA Healthcare System Flow Cytometry Core facility. Sorted live cells were spun for 4min at 300*g*, 4°C, and resuspended to approximately 2,500 cells/ul. Single-cell library preparation was performed by the UCSF Institute for Human Genetics (IHG) and Genomics CoLab core facility by using the 10x Genomics Chromium single cell 5’RNA sequencing platform with feature Barcodes, BCR and TCR library preparation. Sequencing was performed on a NovaSeq6000 with a target depth of 40,000 reads/cell.

Multiplexed CITE-seq libraries from mouse splenocytes were demultiplexed, aligned, and filtered with Cell Ranger, and the aggregated filtered feature-barcode matrix was imported into R for analysis with Seurat. Gene expression counts were used to construct a Seurat object retaining features detected in at least 3 cells and cells expressing at least 200 features, and antibody-derived tag (ADT) features were renamed from oligo identifiers to their corresponding target antigens using the CITE-seq antibody panel annotation. Hashtag oligo (multiplexing capture) counts were added as a separate assay, normalized by centered log-ratio (CLR) transformation, and assigned to samples with HTODemux (positive quantile = 0.99). Cells were quality filtered to exclude those with greater than 5% mitochondrial transcripts, greater than 3% hemoglobin transcripts, and more than 3,500 detected genes, with ribosomal protein and platelet transcript fractions additionally assessed per hashed sample. RNA counts were log-normalized, and variable features were identified, scaled, and reduced by principal component analysis; ADT counts were separately CLR-normalized (per feature), scaled, and reduced by PCA. Weighted-nearest-neighbor integration of the RNA (15 PCs) and ADT (7 PCs) modalities was performed with FindMultiModalNeighbors, followed by UMAP embedding of the weighted graph and clustering of the weighted shared-nearest-neighbor graph using the SLM algorithm at a resolution of 1.2. Residual heterotypic doublets were identified with DoubletFinder using a parameter sweep to select pK = 0.07, pN = 0.25, and an expected doublet rate of 4% adjusted for the homotypic proportion estimated from cluster annotations; predicted doublets were removed, along with cells falling in manually defined high-count/low-complexity regions and cells classified as doublets by hashtag demultiplexing. The resulting singlet object was retained for downstream analysis. Differential expression between comparison groups within each annotated cluster was assessed using the hurdle model implemented in MAST, which jointly models the discrete rate of gene detection and the continuous conditional expression level of detected transcripts. Genes with FDR < 0.05 and |log2 fold change| > 0.2 were considered differentially expressed. Gene set enrichment analysis was performed as described above.

### Immunogold Electron Microscopy (IEM)

Mouse brain tissues for IEM analysis were fixed with 4% PFA overnight. Following the fixation, 40 μm thick free-floating sections were incubated with a cryoprotectant solution for 2 hours at room temperature and freeze-thawed. The sections were incubated with 4% blocking normal goat serum for 1 hour and incubated in 1% blocking normal goat serum overnight. After that, the sections were incubated with 0.2% BSAc and 0.2% fish gelatin (Electron Microscopy Sciences, Hatfield, PA) in PBS for 10 minutes and with gold anti-mouse IgG (H+L), Ultra Small (Electron Microscopy Sciences, 25123, 1:100) for 2 hours. The immunogold particle signal was increased using a silver enhancement kit (Electron Microscopy Sciences, 25521) at 37°C for 2 hours. Finally, the sections were post-fixed in 0.5% osmium tetroxide for 10 minutes, dehydrated, and embedded in resin. To quantify IgG granules, the area within blood vessels were traced using NIH ImageJ, and the number of particles within the blood vessel lumen and in the parenchyma were quantified using Cell Counter.

### snRNA-seq: sample preparation

snRNA seq was performed by microdissecting fresh thalami from *wild type*, *Grn*^*−/−*^*, C9orf72*^*−/−*^, and *Grn*^*−/−*^*;C9orf72*^*−/−*^ mice at 12-month-old (*wild type* (6), *Grn*^*−/−*^ (4), *C9orf72*^*−/−*^ (8), *Grn*^*−/−*^*;C9orf72*^*−/−*^ (6)) as previously described^14,41^. Fresh frozen mouse brain thalami from the left hemisphere were microdissected over ice. They were homogenized in 5 mL of RNase-free lysis buffer (0.32 M sucrose, 5 mM CaCl2, 3 mM Mg(Ac)2, 0.1 mM EDTA, 10 mM Tris-HCl, 1 mM DTT, 0.1% Triton X-100 in DEPC-treated water) using a glass dounce homogenizer (size A; Thomas Scientific) on ice. The homogenate was loaded into a 30-ml polycarbonate ultracentrifuge tube (Beckman-Coulter), and 9 ml of sucrose solution (1.8 M sucrose, 3 mM MgAc_2_, 1 mM DTT and 10 mM Tris-HCl in DEPC-treated water) was carefully added to the bottom of the tube with the homogenate. The tubes were placed in a SW28 rotor and centrifuged at 107,000*g* for 2.5 hours at 4 °C. The supernatant was aspirated, and the nuclei pellets were incubated in 250 mL of DEPC-treated PBS for 20 minutes on ice before resuspending the pellets. Nuclei were counted using a hemocytometer before performing single-nucleus capture on the 10X Genomics Single-Cell 3’ system (version 3.0 and 3.1), following the manufacturer’s instructions. In each sample, 4,000 nuclei were targeted for capture, and 14 cycles thermal cycling were used for cDNA amplification and library amplification. The libraries were sequenced on the NovaSeq 6000 System at the UCSF Genomics Core Facility).

### snRNA-seq: data processing, dimensionality reduction, clustering and UMAP visualization

Gene counts were generated from fastq files using CellRanger software v.7.0.0 by aligning reads to GRC38 genome (mm10). Given three batches of sample preparation, CellRanger Aggr was used to create a unified feature-barcode matrix to aggregate samples and corrects the chemistry differences in v.3.0 and v.3.1 (10x Genomics). Only high quality cells were included for further analysis with the following criteria: number of genes less than 8000, number of RNA molecules per nuclei less than 49,000 and mitochondrial RNAs per cell less than 0.5%. Genes expressed in less than three nuclei were filtered out. DoubletFinder was used to predict doublets which were subsequently filtered out. After applying these quality control steps, the wild type samples yielded 28901 nuclei, 24732 nuclei in *C9orf72*^*−/−*^, 12580 nuclei *Grn*^*−/−*^ and 21438 nuclei *Grn*^*−/−*^*;C9orf72*^*−/−*^. After filtering, the dataset was first batch corrected by SCTransform from Seurat (version 3.4.3), regressing out the number of UMI, mitochondrial gene count, and ribosome count. Subsequently, we performed log normalization on the RNA counts for differential expressed gene analysis and downstream analysis. A UMAP was then used to project the dataset into two-dimensional space with the top 15 principal components. Unbiased clustering was performed with the Louvain–Jaccard method which revealed 16 distinct cell clusters. For sub-clusters in microglia and astrocytes, the clusters were subsetted separately and reclustered using the same method. Using known subtype specific markers, different sub cluster group were identified.

### snRNA-seq: cell type annotation, differential gene expression, and pathway analysis

Cell types were annotated based on expression of known marker genes and using Seurat’s FindAllMarkers function. To identify genes differentially expressed between *C9orf72*^*−/−*^ and *wild type*, *Grn*^*−/−*^ and *wild type*, and *Grn*^*−/−*^*;C9orf72*^*−/−*^ and *wild type*, MAST was used using Seurat’s FindMarkers function. Differentially expressed genes with *P* value greater than 0.05 were filtered out and pathway analysis was performed using ClusterProfiler which was used to generate GO and KEGG pathways. The G scores (Enrichment Score multiplied by −Log_10_(*P*-value)) were used to identify enriched pathways.

### snRNA-seq: gene burden analysis and gene regulatory network analysis

The DEGs were filtered to have Log_2_fold change above 0.25 and FDR below 0.05. Gene burden scores were calculated as the total number of DEGs over the total number of genes expressed in each cluster. This value was normalized to every 1000 genes expressed to normalize the different number of total genes expressed in each cluster. Each data point represents a pairwise comparison between the average control sample and each sample in each genotype. Gene regulatory network analysis was performed using GENIE3 (v1.26.0) to predict the regulatory effect of *Tlr7*. By pseudobulking the cells in each genotype, genes from pathways of interests, including phaogosome and toll-like receptor pathway genes (as shown in Figure 2g), were used as regulators for GENIE3 analysis. The inference weight threshold (weight > 0.03) of *Tlr7* as a regulator was calculated using an elbow plot which was applied to both microglia and astrocyte groups. The expression level of each gene, as indicated by node size, was calculated using the AverageExpression function from Seurat.

### B cell stimulation

Splenocytes from 4-month-old *wild type*, *C9orf72*^*−/−*^*, and Grn*^*−/−*^ and *Grn*^*−/−*^*;C9orf72*^*−/−*^ mice were used to isolate B cells using MACS B cell isolation kit II (Miltenyi Biotec, Cat# 130-104-443). B cell receptor ligation was performed using anti-CD40 (Miltenyi Biotec, Cat# 130-093-022, 5 μg/ml), IgM Fab’ (Jackson ImmunoResearch Laboratories, Cat# 115-006-075, 10 μg/ml), and anti-CD19 antibody (Thermo Fisher, Cat# 14-0194-82, 5 μg/ml). Stimulation of B cells was also performed using R848 (Resiquimod, InvivoGen, Cat# tlrl-r848, 1 μg/ml), 2′3′-cGAMP (InvivoGen, Cat# tlrl-nacga23–02, 5 μg/ml), or CpG2395 (InvivoGen, Cat# tlrl-2395–1, 0.2 μM).

### ELISA

Glutathione-coated plates were prepared by adding 15 ng/100 μL per well of ROBO3 recombinant protein (Abnova/Fisher Scientific, Cat# 89-013-391) in SuperBlock buffer to each well, except blank wells. Plates were incubated for 1 hour at room temperature or overnight at 4°C with mixing. Plates were washed three times with wash buffer and optionally blocked with 100 μL SuperBlock for 1–2 hours. Sera were diluted 1:50 or 1:10, followed by serial dilutions (1:50 to 1:3200) of anti-Robo3 antibodies. After adding 90 μL of SuperBlockT20 and 10 μL of the diluted sample (total dilution of sera 1:500, total dilution of Human CSF 1:10), samples were incubated at room temperature for 1 hour, with optional plate mixing. Plates were washed, and 100 μL of 1:5000 diluted secondary antibodies (Thermo Fisher; Cat#31460, Sigma-Aldrich; AP127P, Invitrogen; 31413) were added. After another hour of incubation, plates were washed, and 50 μL TMB substrate was added, followed by 50 μL of STOP buffer after 10 minutes. Plates were read using SpectraMax iD3 at 450nm.

### Western Blotting

Concentrated recombinant mouse Robo3 Fc chimera proteins (amino acids 54–545, R&D Systems, Cat# 3155-RB, estimated molecular mass 100–110 kDa on SDS-PAGE) was loaded onto SDS-PAGE gels (15 ng/lane). Proteins were transferred to PVDF or nitrocellulose membranes, followed by blocking in buffer for 1 hour and incubation with primary antibodies overnight at 4°C. After washing, membranes were incubated with secondary antibodies, and signals were developed as per the manufacturer’s instructions.

### Phage Immunoprecipitation Sequencing (PhIP-seq)

PhIP-seq was performed using a high-throughput protocol as previously described^59,60^. Briefly, 96-well, 2-ml, deep-well polypropylene plates were incubated with a blocking buffer (3% BSA in TBST) overnight at 4 °C to prevent non-specific binding. Blocking buffer was then replaced with 500 μl of freshly grown phage library (at 1011 plaque-forming units per milliliter) and 1 μl of mouse sera in 1:1 storage buffer (PBS supplemented with 0.04% NaN3, 40% glycerol, 40 mM HEPES). Sera from 12-month-old *wild type* (female n=4, male n=4), *C9orf72*^*−/−*^ (female n=5, male n=2)*, Grn*^*−/−*^ (female n=4, male n=4), and *Grn*^*−/−*^*;C9orf72*^*−/−*^ (male n=6, female n=6) mice were used. To facilitate antibody-phage binding, the deep-well plates with library and sample were incubated overnight at 4°C on a rocker platform in secondary containment. Then, 20 μl of each of Pierce Protein A and G Beads (Thermo Fisher Scientific, 10002D and 10004D) slurry were aliquoted per reaction and washed three times in TNP-40 (140 mM NaCl, 10 mM Tris-HCL, 0.1% NP40). After the final wash, beads were resuspended in TNP-40 in half the slurry volume (20 μl) and added to the phage-patient antibody mixture and incubated on the rocker at 4 °C for 1 hour. Beads were then transferred to a vacuum manifold compatible filter plate65 and then washed five times with RIPA (140 mM NaCl, 10 mM Tris-HCl, 1% Triton X, 0.1% SDS) using an Integra VIAFLO 96 to add RIPA each time and using the vacuum to remove the supernatant. In each wash, beads were incubated for 5 min with gentle rocking at 4 °C. After the fifth wash, immunoprecipitated solution was resuspended in 150 μl of LB-Carb and then added to 0.5 ml of log-phase BL5403 E. coli for amplification (OD600 = 0.4–0.6) until lysis was complete (2 hours) on an 800-r.p.m. shaker. After amplification, sterile 5 M NaCl was added to lysed E. coli to a final concentration of 0.5 M NaCl to ensure complete lysis. The lysed solution was spun at 3,220 relative centrifugal force (rcf) for 20 min, and the top 500 μl was filtered through 0.2 mM PVDF filter plates (Arctic White, AWFP-F20080) to remove remaining cell debris. Filtered solution was transferred to a new pre-blocked deep-well plate where patient sera were added and subjected to another round of immunoprecipitation and amplification. The final lysate was spun at 3,220 rcf for 30 min, with supernatant then filtered and stored at 4 °C for subsequent next-generation sequencing (NGS) library prep. Phage DNA from each sample was barcoded and amplified (Phusion PCR, 18 rounds) and sequenced on an Illumina NovaSeq instrument at an average read depth of 1 million reads per sample.

### CDI Proteome Microarrays

Serum samples from 12-month-old *wild type*, *C9orf72*^*−/−*^*, Grn*^*−/−*^, and *Grn*^*−/−*^*;C9orf72*^*−/−*^ mice were run on HuProt Human Proteome Microarrays (>19,000 human proteins) by CDI Laboratories. Raw serum IgG signal intensities were processed across staining cohort via interquartile range normalization. Antibodies positivity is defined as individual sample signal >3.5x median of the background IgG and >1.5x median of *wild type* IgG signal with a 100% specificity compared to *wild type*. Non-parametric (Wilcoxon) tests were used to compare *C9orf72*^*−/−*^*, Grn*^*−/−*^, and *Grn*^*−/−*^*;C9orf72*^*−/−*^ to *wild type* and corrected with BH test.

### Robo3 ECD immunization protocol

The Robo3 extracellular domain (ECD) immunization protocol involves a 10-week injection of vehicle-only (control) or adjuvant containing mouse Robo3 ECD (designed using NCBI reference sequences: NP_001158239.1 and NM_001164767.1, and UniProt ID: Q9Z2I4 and recombinantly produced in TunaCHO cells at Curia Biologics). The recombinant mouse Robo3 ECD encompasses amino acid 21 to 869, which includes the 5 Ig domains and 3 fibronectin domains, and has a His tag at the C-terminus. On week 0, 4-month-old *wild type*, *C9orf72*^*−/−*^, *Grn*^*−/−*^, and *Grn*^*−/−*^*;C9orf72*^*−/−*^ mice were intraperitoneally injected with 200 μl of a 1:1 emulsion of complete Freund's adjuvant, which included heat-inactivated *Mycobacterium tuberculosis* (HI-TB), with PBS or PBS containing 100 μg of recombinant Robo3 ECD, which was custom-made by Curia. From week 2 to week 10, these 4-month-old *wild type*, *C9orf72*^*−/−*^, *Grn*^*−/−*^, and *Grn*^*−/−*^*;C9orf72*^*−/−*^ mice received a booster immunization every 2 weeks that consisted of 100 μg of Robo3 ECD emulsified 1:1 with incomplete Freund's adjuvant or incomplete Freund’s adjuvant alone (control). Peripheral blood was collected every two weeks via submental blood draw. Animals exhibiting severe immunization-related symptoms were euthanized according to approved humane endpoints. On week 10, the brains of all mice were collected for histopathology analyses.

### TLR7 antagonist treatment

TLR7 antagonist M5049 was purchased from InvivoGen (Cat# inh-m5049) and prepared based on published procedures^35^. Briefly, 0.1M Na-citrate (pH 3.0) was added to one vial of M5049 in powder form (5 mg) for a final concentration of 0.3 μg/μl. The working solution was stored at 4°C, and fresh solution was made every 2 days. The drug was delivered via daily oral gavage using a large bore needle gauge (18–20G) to 6.5-month-old *wild type*, *C9orf72*^*−/−*^, *Grn*^*−/−*^, and *Grn*^*−/−*^*;C9orf72*^*−/−*^ mice for 8 weeks at the dosage of 1μg/gm of body weight (~100 μl per mouse). At the conclusion of the treatment, mice were euthanized using isoflurane overdose and peripheral blood, internal organs, and brains were collected for subsequent analyses, including multi-parameter flow cytometry, measurement of M5049 in brain tissues, and histopathology. The cerebellum was fresh frozen and used to measure the concentration of M5049 in brain tissues by Meadowhawk Biolabs.

### Quantification of IgG using confocal microscopy

Quantification of IgG extravasation and its colocalization with microglia and astrocytes were performed using Fiji. Regions of interest (ROI), which excluded tissue artifacts and channel bleed through, were manually outlined and added to ROI Manager. The nd2 file images were split into individual channels corresponding to IgG, Iba1, and GFAP. For each channel, a maximum intensity projection was generated from z-planes 1–7 for all images. Fixed intensity thresholds were applied to create binary masks (IgG: 1600; Iba1: 1000; GFAP: 1100). Using these thresholds, all the ROIs were and the remaining positive signal was converted to a selection, and the total immunoreactive area was measured. To calculate the IgG per unit area, this value was divided by the total area of each image after excluding the ROIs.

To calculate the colocalization of IgG and Iba1 or GFAP, logical AND operation was used between the corresponding binary masks to generate overlap images. The ROIs to be excluded were similarly removed from the overlap masks before measuring the overlapping area. The extent of IgG colocalization with Iba1^+^ area and GFAP^+^ area was expressed as the ratio of the overlap area to the total Iba1 positive area (IgG∩Iba1/Iba1) or GFAP positive area (IgG∩GFAP/GFAP), respectively.

### Quantification of Synaptophysin^+^ synaptic density

Images were captured using Nikon C2 confocal microscope using a 60x objective, zoom x1, and 8 z-steps, and analyzed using Fiji. FITC-positive signal (Synaptophysin) was identified by splitting image channels and applying a threshold (low: 80 and high:255). Regions of interests (ROIs), excluding tissue artifacts, were defined and saved in the ROI Manager. FITC-positive particles were quantified using *Analyze Particles* with a size range of 10–400 pixels^2^ and circularity of 0.00–1.00. The count was divided by the total area of each image after excluding ROIs. Routinely, 5 images were captured from the ventral thalamus of each mouse, and 5 mice were used in each condition. Statistics were performed using two-way ANOVA with Šídák's Test.

### Statistics and Reproducibility

No statistical methods were used to predetermine the sample size. The data normality distribution was individually tested with the Shapiro-Wilk normality test. Normally distributed datasets were analyzed with a parametric test, while all other analyses were done with a non-parametric approach. All experimental animals were randomly assigned to the treatment or control groups with matching gender and age. For all statistical analysis, a *P* or multi-testing corrected q-value less than 0.05 was considered significant. While no formal statistical methods were used to predetermine sample sizes for all experimental studies, our sample sizes are similar to those reported in our previous publications. No animals or data points were excluded from the analyses. Data collection and analysis were performed blinded to the conditions of the experiments and to the observers. The exact numbers of samples, including images and biological replicates, are indicated either in the figure legends or within graphs. Statistical analyses and graphs were done using Prism 10 (GraphPad). For comparisons between two groups, if normally distributed, two-tailed unpaired Student’s *t*-tests were performed, otherwise, Wilcoxon-tests were used. For multiple group variance comparison, ANOVA tests were performed with normally distributed data, Kruskal-Wallis tests were performed if the data was not normally distributed. Rank-based non-parametric correlation approaches (Spearman’s correlation coefficients) were performed for both continuous and semi-continuous/ordinal variables. For PhIP-seq, raw sequencing reads were aligned to a reference library using Bowtie 2^60^. To correct for differences in sequencing depth, read counts for each peptide within a sample were normalized to the total reads expressed as reads per 100,000 (RPK). FC for each peptide was generated from the mean RPK of the controls, and a z-score was calculated from the background distribution.

### Software usage

The following software and R packages were used in this study: data collection: Leica TCS SP8, data analysis: Fiji (v2.0.0-rc-69/1.52i), IMARIS (v9.8), GraphPad Prism (v10.1.1), FlowJo (v10.9.0), R (v4.1.0), Rstudio (v1.1.463), MultiNicheNet (v1.1.0), DESeq2 (v1.32.0), STAR (v2.7.2.b), edgeR (v3.34.1), GSEA (v4.2.3), CellRanger (v.7.0.0), Seurat (v.4.3.0), iGraph (v2.0.3), clusterProfiler (v4.12.6), dbscan (v1.2), dplyr (v1.1.4), eulerr (v7.0.2), ggplot2 (v3.5.1), limma (v3.60.6), corrplot (v0.92), ggcorrplot (v0.1.4.1), and GENIE3 (v3.19.0).

## Supplementary Material

Supplementary Files

This is a list of supplementary files associated with this preprint. Click to download.

- SupplementaryTable1CITESeqAbs.xlsx
- SupplementaryTable8BrainBulkRNAseq.xlsx
- SupplementaryTable7HumanCSFdeidentified.xlsx
- SupplementaryTable5CDIsignificantantibodies.xlsx
- SupplementaryTable3BcellStimDEGs.xlsx
- SupplementaryInformationGuide.pdf
- SupplementaryTable4PhIPseq.xlsx
- SupplementaryTable10snRNAseqDEGsandpathways.xlsx
- SupplementaryTable6Sharedautoantigens.xlsx
- SupplementaryTable9snRNAseqmetadata.xlsx
- ExtendedDataFiguresXLegends20260924FINAL.pdf
- SupplementaryTable2CITESeqDEGs.xlsx

## Figures and Tables

**Figure 1.**
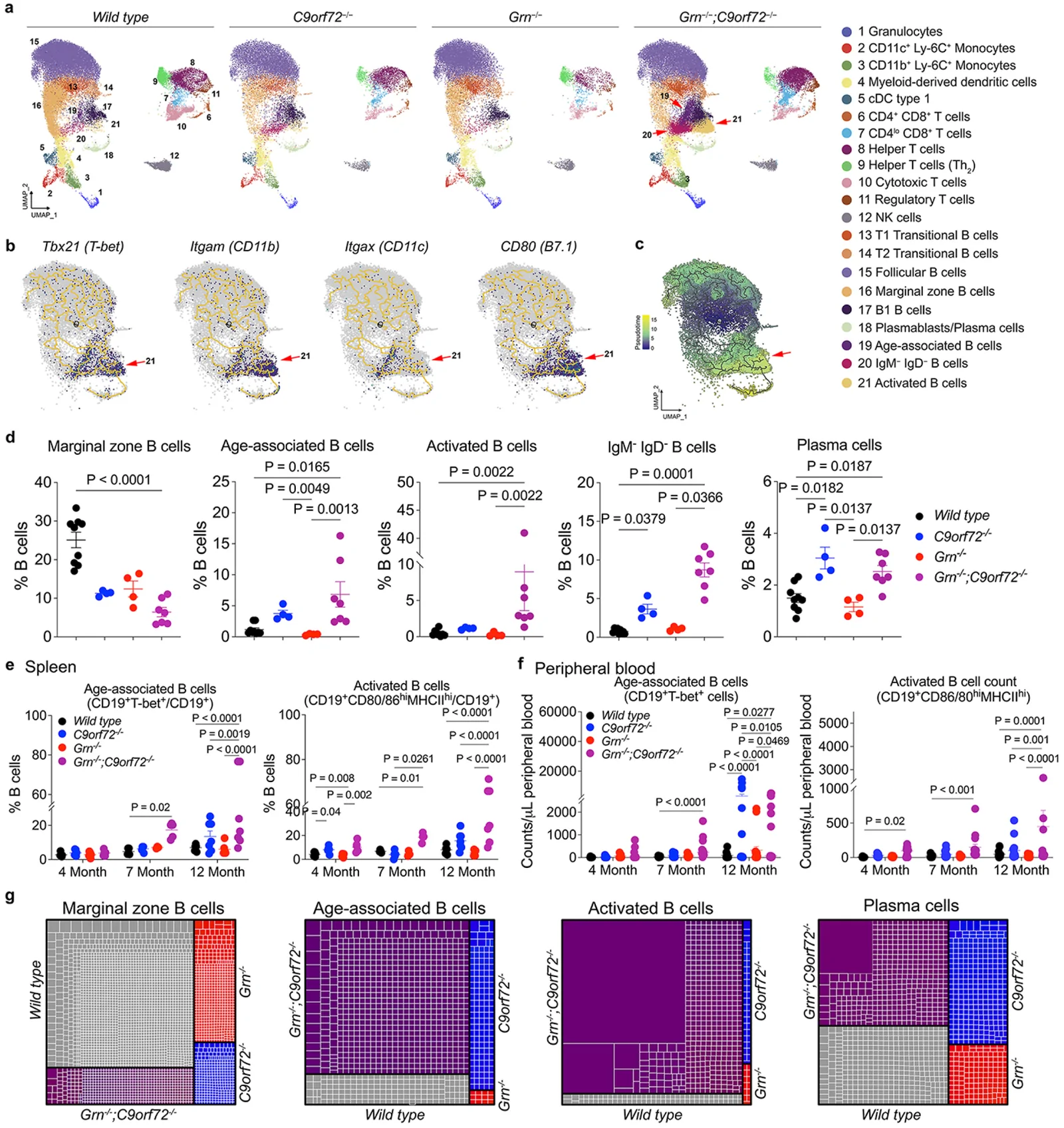
Loss of *Grn* and *C9orf72* promotes age-dependent expansion of B cell subsets. **a.** UMAPs of CITE-seq analyses on the splenocytes from 7-month-old *wild type*, *C9orf72*^*−/−*^, *Grn*^*−/−*^, and *Grn*^*−/−*^*;C9orf72*^*−/−*^ mice (n=4 per genotype, 2 males and 2 females per genotype), shows a significant expansion of age-associated B cells (c19), IgM^−^IgD^−^ B cells (c20), activated B cells (c21), and plasma cells/plasmablasts (c18), but a reduction in marginal zone B cells (c16) in *Grn*^*−/−*^*;C9orf72*^*−/−*^ compared to *wildtype, Grn*^*−/−*^, *C9orf72*^*−/−*^. **b.** Feature plots showing the enriched expression of *Tbx21* (*T-bet*), *Itgam* (*CD11b*), *Itgax* (*CD11c*), and *CD80* (*B7.1*) in age-associated B cell (c20) and activated B cell (c21, arrows) clusters. **c.** Pseudotime analysis shows the aberrant expansion of *T-bet*^+^ age-associated B cells contributes to the expansion of activated B cells and plasma cells. **d.** Multi-parameter flow cytometry of splenic B cells shows a significantly increased abundance of age-associated and activated B cells in *Grn*^*−/−*^*;C9orf72*^*−/−*^ mice compared to *wildtype*, *Grn*^*−/−*^*, C9orf72*^*−/−*^ mice, whereas the relative abundance of marginal zone B cells is decreased in *Grn*^*−/−*^*;C9orf72*^*−/−*^ mice. Increases in the relative abundance of plasma cells are detected in *C9orf72*^*−/−*^ and *Grn*^*−/−*^*;C9orf72*^*−/−*^ mice. **e-f.** Flow cytometry of the splenic B cells and peripheral blood shows an age-dependent increase of age-associated B cells and activated B cells in *Grn*^*−/−*^*;C9orf72*^*−/−*^ mice. Data in panels e-f were from 6 independent biological replicates (n=6) at 4-month-old, n = 10 at 7-month-old, and n = 15 at 12-month-old. Statistics used non-parametric (Wilcoxon test) ranked based test with Benjamani-Hochberg multiple testing correction. **g.** B cell receptor (BCR) repertoire analysis shows that the increased abundance of age-associated B cells, activated B cells, and plasma cells was associated with clonality restriction (age-associated B cells) and oligoclonal expansion (activated B cells and plasma cells).

**Figure 2.**
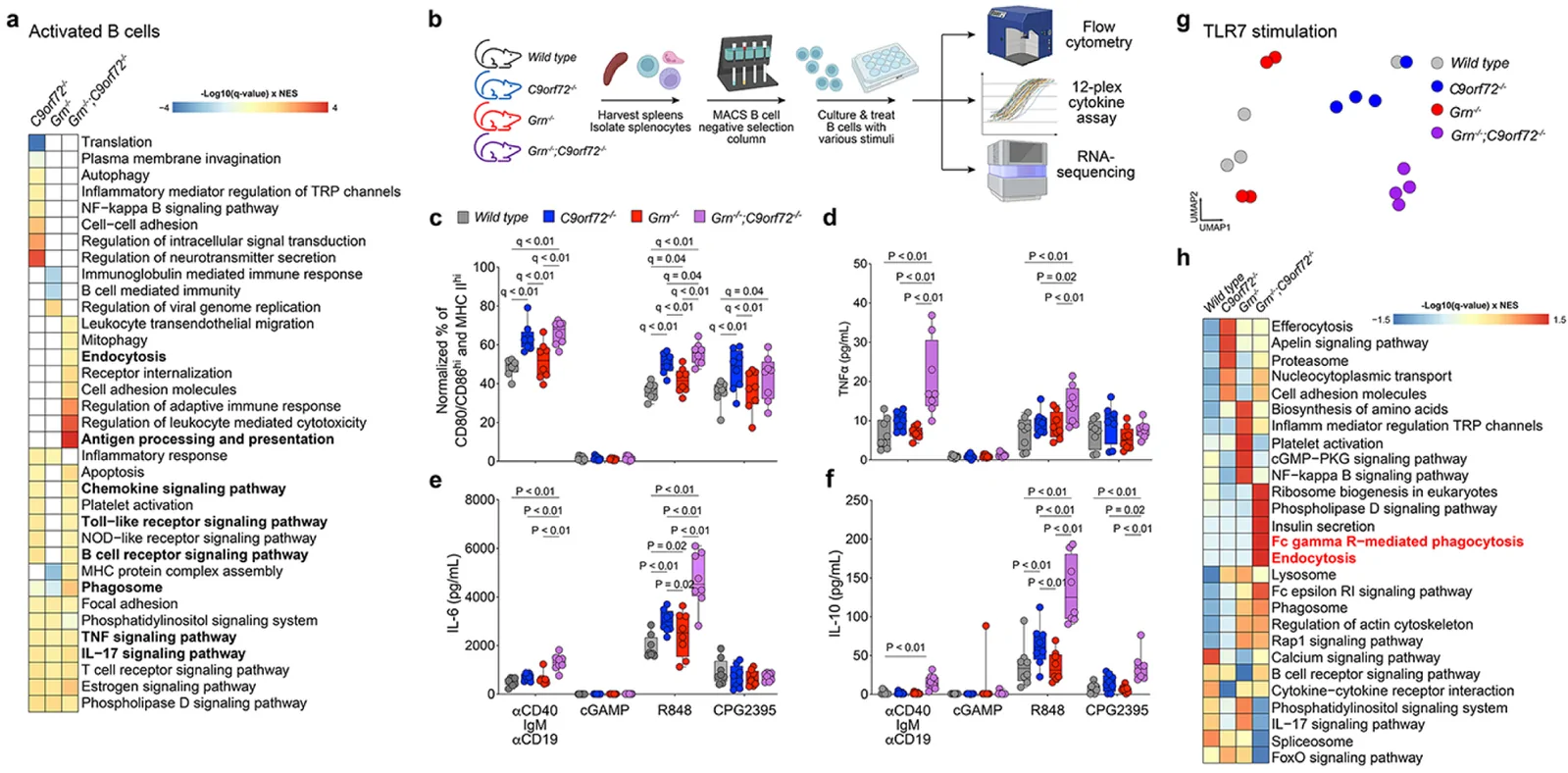
Transcriptomic analyses and cytokine profiling reveal hypersensitivity in *Grn*^*−/−*^*;C9orf72*^*−/−*^ B cells to BCR ligation and toll-like receptor 7 (TLR7) stimulation. **a.** Gene Set Enrichment Analysis (GSEA) of differentially expressed genes (DEGs) revealed significantly upregulated toll-like receptor signaling, NOD-like receptor signaling, phagosome, B cell receptor signaling, and antigen presentation pathways in activated B cells in *Grn*^*−/−*^*;C9orf72*^*−/−*^ mice compared to those from *wild type*. **b.** A schematic diagram showing the experimental plans to isolate, stimulate, and characterize the splenic B cells from 4-month-old *wildtype*, *Grn*^*−/−*^*, C9orf72*^*−/−*^ and *Grn*^*−/−*^*;C9orf72*^*−/−*^ mice using multi-parameter flow cytometry, 12-plex cytokine assays, and bulk RNA-seq. **c.** *In vitro* studies demonstrated that *C9orf72*^*−/−*^ and *Grn*^*−/−*^*;C9orf72*^*−/−*^ B cells and expansion in CD80/86^hi^;MHCII^hi^ cells upon BCR ligation (αCD40/IgM/αCD19 treatment) and TLR7 agonist R848 treatment compared to *wild type* and *Grn*^*−/−*^ B cells. Stimulation of the STING pathway has no effects in the expansion of CD80/86^hi^;MHCII^hi^ cells in any of the 4 genotypes. **d-f**. BCR ligation and treatment with TLR7 agonist consistently increase the secretion of pro-inflammatory cytokine TNFαμ(panel d), IL-6 (panel **e**) and IL-10 (panel **f**), though TLR7 agonist has most robust effects in IL-6 and IL-10. In contrast, treatment with TLR9 agonist modestly increases the secretion of IL-10 in *Grn*^*−/−*^*;C9orf72*^*−/−*^ splenic B cells. Data in panels d-f were from 8 independent biological replicates. Statistics used non-parametric (Wilcoxon test) ranked based test with Benjamani-Hochberg multiple testing correction. **g**. UMAP showing genotype-specific changes in transcriptomic profiles as revealed by bulk RNA-seq in *wild type*, *Grn*^*−/−*^, and *Grn*^*−/−*^*;C9orf72*^*−/−*^ B cells upon treatment with TLR7 agonist. **h.** A heatmap summarizing the enriched GO terms and KEGG pathways in *wild type*, *Grn*^*−/−*^, and *Grn*^*−/−*^*;C9orf72*^*−/−*^ B cells after TLR7 stimulation, with *Grn*^*−/−*^*;C9orf72*^*−/−*^ B cells showing a genotype-specific increased in Fc gamma R-mediated phagocytosis and endocytosis.

**Figure 3.**
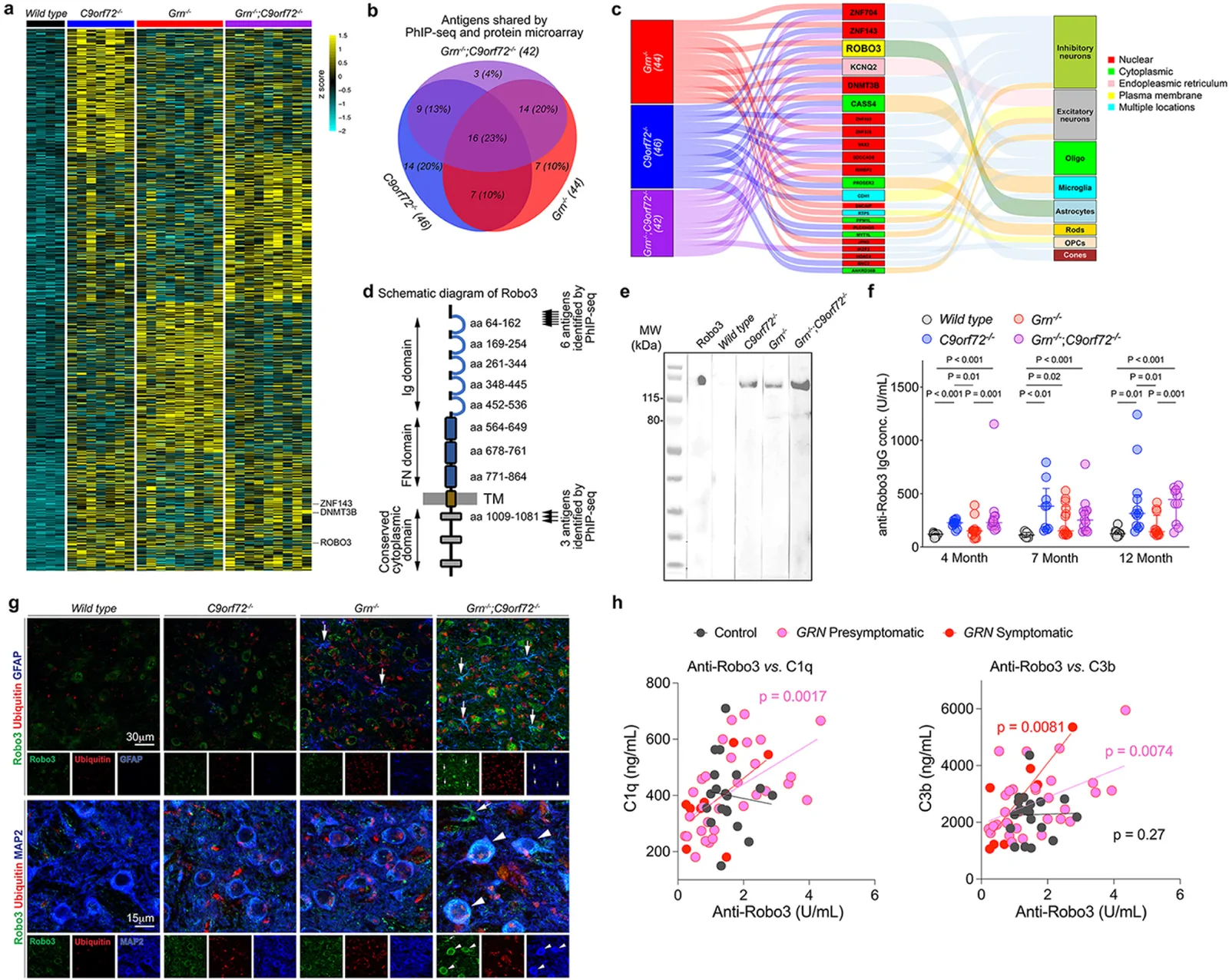
Autoantibody profiling reveals Robo3 as a CNS autoantigen target in *Grn*^*−/−*^*, C9orf72*^*−/−*^*, and Grn*^*−/−*^*;C9orf72*^*−/−*^ mice. **a.** CDI whole proteomic array and PhIP-seq identify genotype-specific and shared autoantibodies in sera from 12-month-old *Grn*^*−/−*^*, C9orf72*^*−/−*^*, and Grn*^*−/−*^*;C9orf72*^*−/−*^ mice. **b.** A Venn diagram showing 70 autoantigens identified by CDI and PhIP-seq assays shared by *Grn*^*−/−*^*, C9orf72*^*−/−*^*, and Grn*^*−/−*^*;C9orf72*^*−/−*^ mice. **c.** Sankey plots showing the CNS-related autoantigens based on the genotypes (left column), subcellular localizations (middle column), and cell types (right column). This approach reveals that Robo3 is the only plasma membrane protein targeted by autoantibodies detected in *Grn*^*−/−*^*, C9orf72*^*−/−*^*, and Grn*^*−/−*^*;C9orf72*^*−/−*^ sera. **d.** PhIP-Seq identifies 6 antibody-binding epitopes near the first Ig domain of Robo3 and 3 epitopes in the conserved cytoplasmic domain. **e.** Western blots showing the presence of anti-Robo3 autoantibodies in sera from 4-month-old *Grn*^*−/−*^*, C9orf72*^*−/−*^*, and Grn*^*−/−*^*;C9orf72*^*−/−*^ mice. **f.** Enzyme-linked immunosorbent assays (ELISA) using recombinant Robo3 proteins also detect the presence of anti-Robo3 autoantibodies in sera from 4, 7 and 12-month-old *Grn*^*−/−*^*, C9orf72*^*−/−*^*, and Grn*^*−/−*^*;C9orf72*^*−/−*^ mice. Data were from 10 independent biological replicates. Statistics used non-parametric (Wilcoxon) rank-based test with Benjamani-Hochberg multiple testing correction. **g.** Immunohistochemical stains of Robo3 and GFAP (top panel) and Robo3 and MAP2 (lower panel) show increased Robo3 expression in GFAP^+^ astrocyte and MAP2^+^ neurons in *Grn*^*−/−*^*;C9orf72*^*−/−*^ thalamus. **h.** Correlation plots showing that anti-ROBO3 autoantibody titers in presymptomatic and symptomatic *GRN* carriers and its correlation with complements C1q and C3b levels.

**Figure 4.**
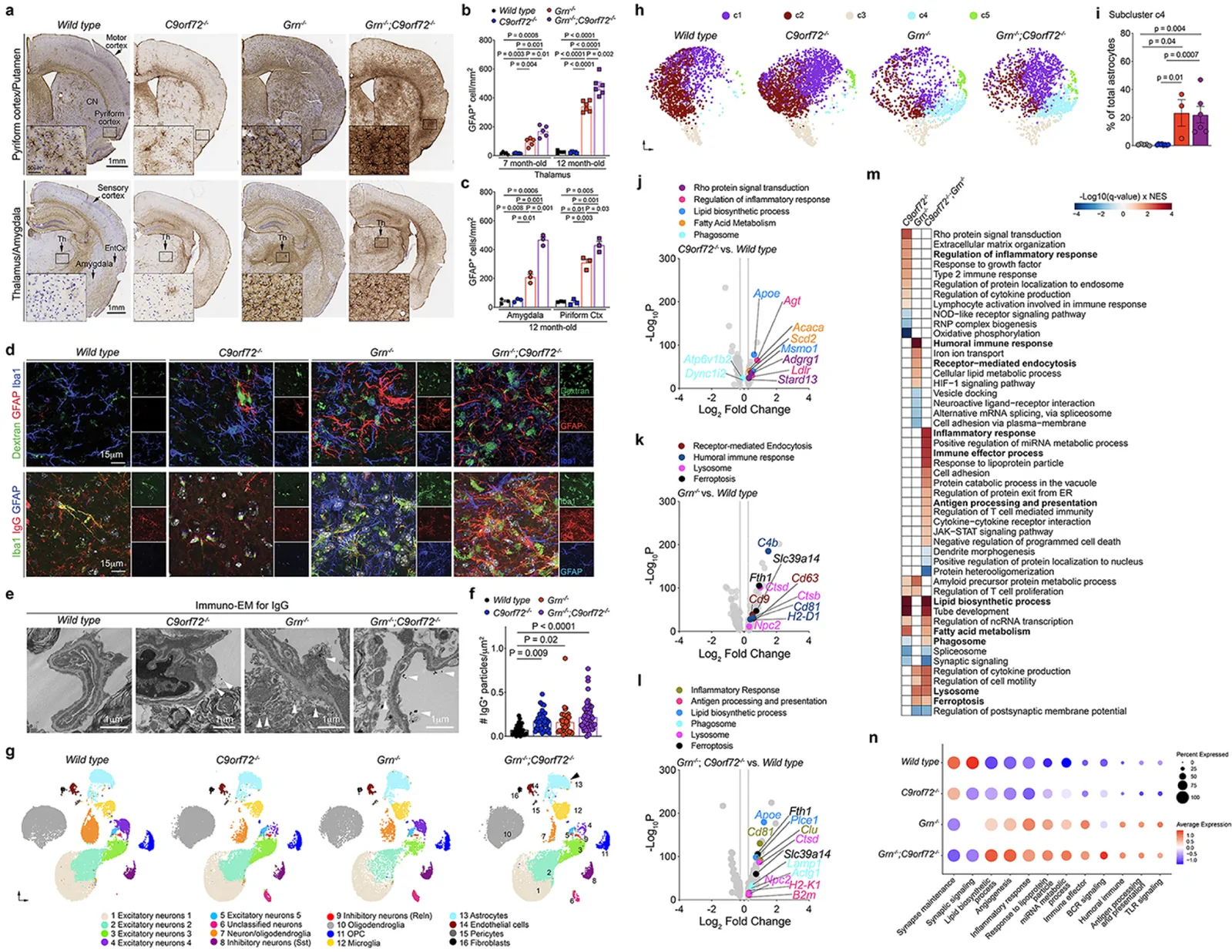
Severe astroglial pathology in *Grn*^*−/−*^*;C9orf72*^*−/−*^ mice. **a.** Immunohistochemical stains showed a higher density of GFAP^+^ astrocytes in the thalamus, amygdala, and pyriform cortex of 12-month-old *wild type*, *C9orf72*^*−/−*^*, and Grn*^*−/−*^ and *Grn*^*−/−*^*;C9orf72*^*−/−*^ mouse brains. **b-c.** Stereological quantification of GFAP^+^ astrocyte density in the thalamus at 7 and 12-month-old (panel **b**) and amygdala and piriform cortex at 12-month-old (panel **c**) of *wild type*, *C9orf72*^*−/−*^*, Grn*^*−/−*^ and *Grn*^*−/−*^*;C9orf72*^*−/−*^ mice. Statistics used one-way ANOVA with Tukey’s multiple comparison test. **d.** Confocal images show the presence of 40 kDa FITC-conjugated Dextran and IgG in Iba1^+^ microglia and GFAP^+^ astrocytes in the thalamus of 12-month-old *Grn*^*−/−*^ and *Grn*^*−/−*^*;C9orf72*^*−/−*^ mice. **e.** Immunogold electron microscopy for IgG outside the basement membrane of endothelial cells in the thalamus of 12-month-old *C9orf72*^*−/−*^*, Grn*^*−/−*^, and *Grn*^*−/−*^*;C9orf72*^*−/−*^ mice. **f.** Quantification of IgG^+^ immunogold particles outside the basement membrane of endothelial cells in the thalamus of 12-month-old *wild type*, *C9orf72*^*−/−*^*, Grn*^*−/−*^, and *Grn*^*−/−*^*;C9orf72*^*−/−*^ mice (n=3, 10 images per mouse). Statistics used one-way ANOVA with Tukey’s multiple comparison test. **g.** Unsupervised clustering of snRNA-seq data from the thalamus of 12-month-old *wild type*, *C9orf72*^*−/−*^, *Grn*^*−/−*^ and *Grn*^*−/−*^*;C9orf72*^*−/−*^ mice reveal 16 unique clusters. **h.** UMAP of astrocyte subclusters. **i.** Quantification of astrocyte subclusters in *wild type*, *C9orf72*^*−/−*^, *Grn*^*−/−*^ and *Grn*^*−/−*^*;C9orf72*^*−/−*^ thalamus show an expansion of cluster c4 in *Grn*^*−/−*^ and *Grn*^*−/−*^*;C9orf72*^*−/−*^ thalamus compared to *wild type* and *C9orf72*^*−/−*^ thalamus. **j-l.** Volcano plots show differentially expressed genes (DEGs) identified in *C9orf72*^*−/−*^, *Grn*^*−/−*^ and *Grn*^*−/−*^*;C9orf72*^*−/−*^ astrocytes compared to *wild type* astrocytes. P values and fold changes were calculated using MAST and highlighted genes belong to color-matched pathways in each volcano plot. The genes were filtered using *P* < 0.05 and above 1.2 fold change (highlighted by dashed line). **m.** GSEA using ClusterProfiler reveal GO terms and KEGG pathways enriched in *C9orf72*^*−/−*^, *Grn*^*−/−*^ and *Grn*^*−/−*^*;C9orf72*^*−/−*^ astrocytes compared to those from *wild type* mice. **n.** Module score using GO pathways of interest measuring the relative average expression of specific pathway and gene set across 4 genotypes.

**Figure 5.**
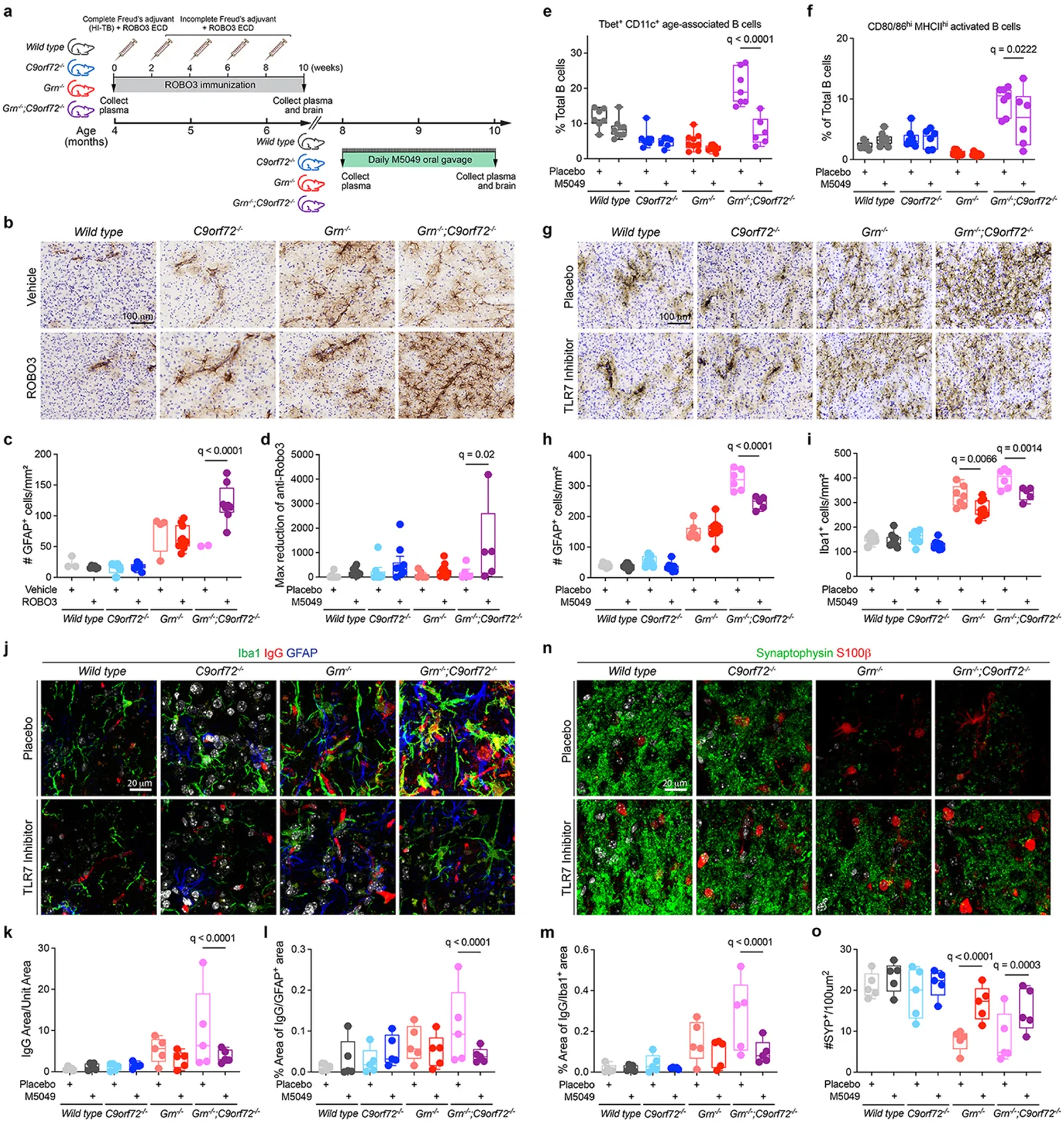
Immunization with Robo3 ECD promotes, whereas treatment with TLR antagonist M5049 rescues astrogliosis in *Grn*^*−/−*^*;C9orf72*^*−/−*^ mice. **a.** Schematic diagrams for a 10-week Robo3 active immunization protocol using Robo3 extracellular domain (ECD) in 4-month-old *wild type*, *C9orf72*^*−/−*^, *Grn*^*−/−*^ and *Grn*^*−/−*^*;C9orf72*^*−/−*^ mice or an 8-week TLR7 inhibition protocol with daily oral gavage of TLR7 antagonist M5049 starting at 8-month-old in *wild type*, *C9orf72*^*−/−*^, *Grn*^*−/−*^ and *Grn*^*−/−*^*;C9orf72*^*−/−*^ mice. **b-c.** Immunohistochemical stains of GFAP^+^ astrocyte show increased astrogliosis in *Grn*^*−/−*^*;C9orf72*^*−/−*^ after Robo3 immunization. **d.** ELISA using recombinant Robo3 protein showed a reduction of anti-Robo3 autoantibody titers in plasma from *Grn*^*−/−*^*;C9orf72*^*−/−*^ mice after M5049 treatment. **e.** Multi-parameter flow cytometry shows significant reduction in Tbet^+^CD11c^+^ age-associated B cells and CD80/86^hi^MHC^hi^ activated B cells in *Grn*^*−/−*^*;C9orf72*^*−/−*^ mice compared to *wild type*, *Grn*^*−/−*^, and *C9orf72*^*−/−*^ mice. **g-i.** Immunohistochemical stains of GFAP^+^ astrocyte show a significant reduction in GFAP^+^ astrocytes in *Grn*^*−/−*^*;C9orf72*^*−/−*^ mice (panels **g**-**h**). In addition, M5049 treatment reduces Iba1^+^ microglia density in *Grn*^*−/−*^ and *Grn*^*−/−*^*;C9orf72*^*−/−*^ mice (panel i). **j-m**. TLR7 antagonist treatment reduces the engulfment of IgG in GFAP^+^ astrocytes and Iba1^+^ microglia in *Grn*^*−/−*^*;C9orf72*^*−/−*^ mice. **n-o.** TLR7 antagonist M5049 also rescues Synaptophysin^+^ presynaptic density in *Grn*^*−/−*^ and *Grn*^*−/−*^*;C9orf72*^*−/−*^ mice. All statistics were performed using two-way ANOVA with Šídák's Test.

## Data Availability

CITE-seq, bulk RNA-seq, and snRNA-seq have been deposited to the Sequence Read Archive under accession no. PRJNA1198091. Source data are provided with this paper.
